# Genome-Wide Association and Selection Signature Analyses of Hematological and Serum Biochemical Traits in Yunling Cattle

**DOI:** 10.3390/ani16203302

**Published:** 2026-10-20

**Authors:** Jiafei Shen, Yanan Wang, Yunxin Dong, Qianlin Lv, Yiming Huang, Yingcan Miao, Quratulain Hanif, Kaixing Qu, Chuzhao Lei

**Affiliations:** 1 Jiangsu Key Laboratory for Bioresources of Saline Soils, Jiangsu Synthetic Innovation Center for Coastal Bio-Agriculture, Jiangsu Provincial Key Laboratory of Coastal Wetland Bioresources and Environmental Protection, School of Wetlands, Yancheng Teachers University, Yancheng 224051, China; shenjiafei0118@163.com (J.S.); momomomo6633@163.com (Y.W.); dongyunxin0415@163.com (Y.D.); 15394688659@163.com (Q.L.); hym18406317429@163.com (Y.H.); yingcanmiao@gmail.com (Y.M.); 2 Department of Life Sciences, School of Science, University of Management and Technology, Lahore 54770, Pakistan; micro32uvas@gmail.com; 3 Academy of Science and Technology, Chuxiong Normal College, Chuxiong 675000, China; kaixqu@cxtc.edu.cn; 4 Key Laboratory of Animal Genetics, Breeding and Reproduction of Shaanxi Province, College of Animal Science and Technology, Northwest A&F University, Yangling 712100, China

**Keywords:** Yunling cattle, blood traits, GWAS, selection signature, candidate genes

## Abstract

Yunling cattle, a native breed native to subtropical regions, exhibit remarkable traits such as heat tolerance, strong immunity, and tick resistance. To characterize the genetic architecture of blood physiological and biochemical traits associated with these adaptations, we performed a genome-wide association study (GWAS) using whole-genome resequencing data from 97 individuals combined alongside 39 blood traits (30 analysed after quality control). Significant association signals were detected for seven traits: albumin (ALB), homocysteine (CYS), white blood cell count (WBC), bilirubin (BIL), indirect bilirubin (I-BIL), low-density lipoprotein cholesterol (LD-CHO), and red cell distribution width (RDW). Notably, loci related to leukocyte parameters overlapped with strong selection signals and were mapped to classical bovine leukocyte antigen (BOLA) genes, including *BOLA*, *BOLA-DQB* and *BOLA-DQA5*, which are located within the MHC region and are known to be involved in immune regulation. For lipid-related traits, the strongest signals were identified near *RPL22L1* and *SLC7A14* on chromosome 1, while additional associated genes included *XPOT*, *SLC16A7*, *SCAI*, *SYT14* and *ATP2B2*. Additionally, regarding metabolic and protein parameters, *NHSL1* showed a strong association with bilirubin; this gene has previously been reported to be associated with hypertension. Red blood cell parameters revealed candidate genes such as *NAV3*, *LSAMP*, *ACACA*, and others linked to erythrocyte variation. For Homocysteine-related traits, *CIITA* was significantly associated with Homocysteine levels; *CIITA* is known to be involved in immune regulation. In conclusion, this study provides a genome-wide characterization of the genetic architecture of blood traits in Yunling cattle, identifying several candidate genes (e.g., *MOB1B*, *CIITA* and *BOLA* genes) associated with blood physiological and biochemical traits. These candidate loci provide a foundation for further functional validation; validation in larger and independent populations is needed before any application.

## Simple Summary

Yunling cattle are a Chinese breed known for their adaptability to subtropical environments and resistance to ticks and diseases. To uncover the genetic basis of their blood physiological and biochemical traits, we analyzed the genomes of 97 cattle and 30 blood metabolic and biochemical parameters. We identified seven blood traits with significant genetic associations. Key genes were found in the bovine leukocyte antigen (BOLA) region, which is known to play a role in immune regulation, while others were associated with metabolism. Notably, the *CIITA* gene was significantly associated with homocysteine levels and is known to be involved in immune regulation. These findings provide candidate loci for the genetic architecture of blood traits in Yunling cattle.

## 1. Introduction

Yunling cattle, a ternary hybrid breed developed over more than 30 years by the Yunnan Academy of Grassland and Animal Science in China, has been recognized for its rapid growth rate, exceptional heat tolerance, strong immunity, and resistance to tick infestation [1]. Previous genomic analyses have revealed that Yunling cattle inherit approximately 40% of their genome from European taurine cattle (e.g., Angus and Simmental), 56% from Indian zebu cattle, and 4% from Chinese zebu cattle [2]. The zebu-derived genomic components are particularly notable, as they are considered to underpin the breed’s adaptive advantages in subtropical environments, including disease resistance and thermotolerance. Zhao et al. further demonstrated that Yunling cattle exhibit significantly lower tick infestation rates and higher blood histamine concentrations compared to exotic breeds such as Angus and Simmental, and that polymorphisms in *TLR4* is associated with these resistance traits [3]. Owing to these superior characteristics, Yunling cattle have become a valuable genetic resource for beef cattle breeding in subtropical China, valued for their adaptability to hot and humid environments, disease resistance, and good growth performance. The breed has been widely used in crossbreeding programmes to improve the productivity of local cattle populations.

Hematological and serum biochemical parameters are reliable indicators of an animal’s physiological and pathological status, reflecting metabolic homeostasis, organ function, and immune competence [4,5]. These traits are routinely monitored in clinical diagnostics and have increasingly been adopted in veterinary medicine for disease surveillance and health management in livestock [6,7,8]. Crucially, accumulating evidence from twin studies and pedigree analyses indicates that most blood parameters including red blood cell indices, white blood cell counts, lipid profiles, and enzyme activities are moderately to highly heritable, with heritability (h^2^) estimates typically ranging from 0.20 to 0.60 in both humans and cattle [9,10,11]. The heritability of blood traits varies across trait categories. Traits under strong homeostatic control (e.g., electrolytes and minerals) tend to have lower heritability, because their concentrations are tightly regulated by physiological feedback mechanisms. In contrast, traits such as white blood cell counts, lipid profiles, and enzyme activities have higher heritability, reflecting stronger genetic determination. This substantial genetic component makes blood parameters tractable phenotypes for genome-wide association studies (GWAS) aimed at uncovering the genetic architecture underlying immune function and disease susceptibility. Over the past decade, GWAS of hematological and biochemical traits in cattle have been primarily conducted in dairy breeds such as Holstein [10,12], focusing on serum cholesterol, liver enzyme activities, and mineral concentrations. However, similar studies in indigenous beef cattle breeds adapted to tropical and subtropical environments remain scarce, limiting our understanding of how natural and artificial selection have shaped their immune-related genomic landscapes.

Selection signature analysis provides a complementary approach to GWAS for identifying genomic regions underlying adaptive traits. While GWAS detects statistical associations between genetic variants and phenotypic variation in contemporary populations, selection signature methods—such as nucleotide diversity (θπ), Tajima’s D, and the Composite Likelihood Ratio (CLR) test—scan for genomic footprints of historical selective pressure [13,14]. Regions that have been targets of natural or artificial selection often exhibit reduced diversity, extended haplotype homozygosity, or skewed allele frequency spectra. Integrating these two strategies offers a powerful framework: GWAS pinpoints trait-associated loci at the population level, while selection signature analysis provides evolutionary evidence that these loci have been functionally important [15]. This integrative approach has been successfully applied in cattle to identify candidate genes underlying production, reproduction, and health traits, yet it has rarely been exploited to dissect the genetic basis of blood parameters in indigenous Chinese beef breeds.

Despite the well-documented immune advantages of Yunling cattle, the genetic basis of their hematological and biochemical phenotypes remains largely unexplored. No comprehensive GWAS or genome-wide selection scan has been conducted to systematically characterize the genetic variants and genomic regions associated with blood traits in this breed. We hypothesized that (i) blood physiological and biochemical traits in Yunling cattle harbor genomic regions associated with blood phenotypes; and (ii) candidate genes underlying these traits overlap with regions under historical selection, providing insight into the genetic basis of the breed’s adaptive advantages. To address this gap, we performed a genome-wide association study and selection signature analysis using whole-genome resequencing data from 97 Yunling cattle. Specifically, we aimed to: (i) characterize the phenotypic variation of 39 blood traits (30 biochemical and 9 haematological); (ii) identify SNPs and genomic regions significantly associated with these immune-related phenotypes through univariate GWAS; (iii) detect signatures of historical selection across the genome using θπ, Tajima’s D, and CLR methods; and (iv) integrate both approaches to prioritize robust candidate genes governing immunity and disease resistance in Yunling cattle.

## 2. Materials and Methods

### 2.1. Samples and Blood Index Measurement

A total of 97 Yunling cattle with available blood physiological and biochemical records were used in this study. Blood samples were collected on the morning of 5 January 2019. These animals were derived from the population whose whole-genome resequencing data were deposited in BioProject PRJNA555741 in July 2019 [15]. The correspondence between the sequenced individuals and the phenotyped animals was verified using ear-tag records and the breeding registry of the core breeding farm. All animals were raised at the core breeding farm of the Yunnan Academy of Grassland and Animal Science. The feeding regime consisted of two management systems: pen-feeding and free-grazing. Pen-fed individuals were kept under uniform feeding and management conditions, while free-grazing individuals grazed on pasture and were supplemented with feed at fixed times and locations. All animals were multiparous adult females approximately 3 years of age. To avoid the influence of reproductive status, animals within 2 weeks pre- or post-calving were excluded from the study.

After the skin of the blood collection site was disinfected with 75% alcohol, blood samples were collected from the jugular vein. For each animal, 10 mL of whole blood was collected into an EDTA tube, 5 mL into a coagulant tube, and 5 mL into a lithium heparin tube. Blood samples were analyzed within 2 h of collection. Hematological and biochemical indices were measured at the laboratory of Yunnan Red Society Hospital. Hematological indices (WBC, RBC, HGB, HCT, MCV, MCH, MCHC, RDW, and PLT) were measured using an automatic hematology analyzer (Sysmex XB-2100, Sysmex Co., Ltd., Kobe, Japan), as previously described in our related study on the same farm population [16]. Biochemical indices were measured using an automatic biochemical analyzer (LABOSPECT 008α, Hitachi High-Tech Corporation, Tokyo, Japan) at the same laboratory. A small number of indices were measured using commercially available kits following the manufacturers’ instructions. The instrument information was provided by the collaborating laboratory.

### 2.2. Genome-Wide Selection Pressure Analysis

Genome scans for selection in Yunling cattle were performed using the following strategy. Firstly, nucleotide diversity (θπ) was calculated using VCFtools (v0.1.17) (--window-pi 50000 --window-pi-step 20000). Secondly, based on the site frequency spectrum, Tajima’s D was estimated using VCFtools (--TajimaD 50000) [13]. This test detects the signatures of selection or demographic events, typically yielding negative values under population expansion, in the absence of selection. Finally, using the empirical frequency spectrum with an allele frequency file combined across all autosomes, the Composite Likelihood Ratio test (CLR) was performed using SweepFinder2 (v1.0) [14]. Windows < 10 SNPs were excluded, and 49,746 windows for θπ and Tajima’s D test were retained. A 50 kb window was chosen to allow comparison and alignment among θπ, Tajima’s D and CLR values. Empirical *p* value for θπ, Tajima’s D and CLR was calculated. For θπ and CLR, the top 0.5% of windows were considered as candidate signatures of selection. For Tajima’s D, because the statistic can take both positive and negative values, the top 0.25% of positive windows and the top 0.25% of negative windows were each considered, together corresponding to the top 0.5% of windows overall, consistent with the thresholds used for θπ and CLR. Functional annotation of the candidate SNPs was performed using ANNOVAR (v0.0.2) [17] based on *Bos taurus* reference genome ARS-UCD1.2. GO and KEGG enrichment analyses were performed using KOBAS (http://bioinfo.org/kobas/, accessed on 13 October 2026) [18]. The candidate gene list was used as input, with the bovine genome as background. Pathways with FDR < 0.05 were considered significantly enriched.

### 2.3. Genome-Wide Association Study Analysis

We performed a univariate GWAS using a linear mixed model to examine the potential associations between 39 blood traits (30 analysed after quality control) and genomic variants using GEMMA (v0.98.5) [19]. The statistical model was as follows:

*y* = *μ* + *Xβ* + *Zu* + *ε*

where *y* denotes the vector of phenotypic values, *μ* is the overall mean, *X* is the design matrix for fixed effects (principal component 1 and feeding regime), *β* is the vector of fixed-effect coefficients, *Z* is the incidence matrix relating individuals to the polygenic random effect, *u* is the vector of polygenic effects with *u* ~ N(0, Kσ^2^_a_), where K is the kinship matrix, and *ε* is the vector of residual errors with *ε* ~ N(0, Iσ^2^_e_). The first principal component (PC1) was included as a fixed effect to control for population structure. PC1 clearly separated the Brahman and Yunling cattle in our previous study [20]; additional principal components (PC2–PC4) were also evaluated but explained minimal additional variance and were therefore not retained. The feeding regime (pen-feeding vs. free-grazing) was included as a fixed effect. Age and sex were not included as covariates because all animals were adult females of approximately the same age (~3 years), so these variables had no variation. Quantile–quantile (QQ) plots were generated for all analysed traits to assess whether the test statistics were well calibrated.

The thresholds were set at *P*_wald_ = 5 × 10^−8^ (genome wide significance) and *P*_wald_ = 1 × 10^−6^ (suggestive) [19]. The 5 × 10^−8^ threshold was originally derived from human genetics and is widely used as a conservative genome-wide significance threshold in cattle GWAS. Because many blood traits are partially correlated, a strict Bonferroni correction across the 30 analysed traits would be overly conservative. We therefore retained 5 × 10^−8^ as the genome-wide significance threshold and 1 × 10^−6^ as the suggestive threshold, and consistently distinguish between genome-wide significant and suggestive associations throughout the manuscript. In this model, principal component 1 and the kinship matrix were included to address the fixed effect of population structure and the random effects of relatedness among individuals. The kinship matrix was calculated using GEMMA based on the filtered SNP set (24.55 million autosomal biallelic SNPs), following the standard approach described by Zhou and Stephens (2012) [19]. Specifically, the centered relatedness matrix was computed as described in the GEMMA documentation. The kinship matrix was included as a random effect in the linear mixed model, with u ~ N(0, Kσ^2^), where K is the kinship matrix.

### 2.4. Correlation and Mantel Test Analysis

Pearson correlation coefficients among the 30 blood parameters retained after quality control were calculated and visualized as a correlation heat map. A Mantel test was performed to assess the correlation between the hematological parameter matrix (white blood cell, red blood cell, and platelet parameters) and the blood biochemical parameter matrix, based on Euclidean distance matrices. The Mantel statistic (r) was calculated in R (v4.3.1) using the mantel function in the vegan package (v2.6_4) with 999 permutations.

## 3. Results

### 3.1. SNP Distribution and Phenotype Statistic After Quality Control

A total of ~10.21 billion clean reads were generated from the published resequencing data of 97 Yunling cattle (Table S1) [2]. The reads were aligned to the *B. taurus* reference genome ARS-UCD1.2 with an average alignment rate of 99.55% and a mean genome coverage of 5.618×. A total of ~24.55 million autosomal biallelic SNPs were retained after filtering out the potential PCR duplicates and false positives.

We also measured 30 blood biochemical traits (*lipid-related parameters*: cholesterol, Chol; high-density lipoprotein cholesterol, HD-Chol; low-density lipoprotein cholesterol, LD-Chol; triglycerides, TG; *metabolism and protein-related parameters*: glucose, GLU; urea ammonia, UAM; uric acid, UAC; total bilirubin, T-Bil; direct bilirubin, D-BIL; indirect bilirubin, I-BIL; total proteins, T-Prot; albumin, ALB; globulin, GLO; prealbumin, PAB; *enzyme activities*: Alkaline phosphatase, ALP; Alanine aminotransferase, ALT; Aspartate aminotransferase, AST; γ-Glutamyl transpeptidase, GLT; Cholinesterase, CLT; creatine kinase, CK; *electrolytes*: serum hydrogen phosphate, SHP; potassium, K; sodium, Na; chlorine, CL; calcium, Ca; phosphorus, P; magnesium, Mg) and 9 hematological traits (*erythrocyte traits*: red blood cell count, RBC; hemoglobin, HGB; hematocrit, HCT; mean corpuscular volume, MCV; mean corpuscular hemoglobin, MCH; mean corpuscular hemoglobin concentration, MCHC; and red cell distribution width, RDW; *leukocyte traits*: white blood cell count, WBC; *platelet traits*: platelet count, PLT) in Yunling cattle. Table 1 presents the descriptive statistics of hematological and biochemical traits, including maximum, minimum, mean, standard deviation, 95% confidence level of the mean, skewness and kurtosis. Traits with a skewness outside the interval from −1 to 1, or with a kurtosis greater than 2, were considered to depart substantially from normality and were excluded from subsequent GWAS analysis [21]. As a result, PALB, AST, GT, ALP, TBC, GLC, CRE, CL and PLT were not used in subsequent GWAS analysis.

**Figure 1:**
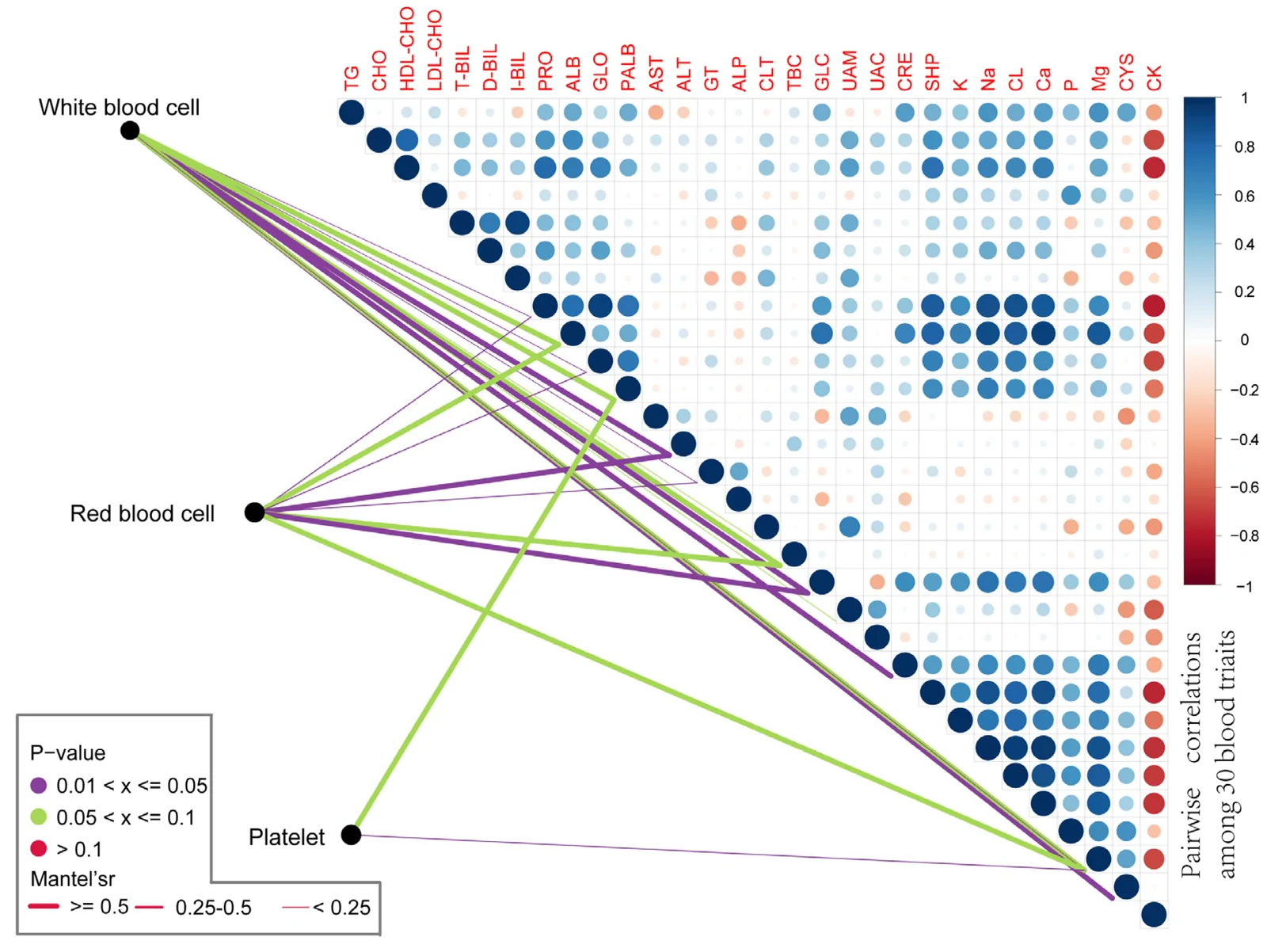
Network representing the Pearson’s correlation coefficients among the 30 blood biochemical traits measured in this study. The heat map shows pairwise correlations among blood traits, and the network links connect hematological parameters (white blood cell, red blood cell, and platelet) to correlated blood biochemical traits. Line colour indicates the significance level: purple, 0.01 < *p* ≤ 0.05; light green, 0.05 < *p* ≤ 0.1; red, *p* > 0.1. Mantel’s r values are indicated by line width: thick, r ≥ 0.5; medium, 0.25 ≤ r < 0.5; thin, r < 0.25. Note that these 30 biochemical traits include several traits (PALB, AST, GT, ALP, TBC, GLC, CRE, CL) that were subsequently excluded from GWAS based on quality-control criteria. Abbreviations: TG, triglycerides; CHO, total cholesterol; HD-CHO, high-density lipoprotein cholesterol; LD-CHO, low-density lipoprotein cholesterol; BIL, total bilirubin; DBIL, direct bilirubin; IBIL, indirect bilirubin; PRO, total proteins; ALB, albumin; GLO, globulin; PALB, prealbumin; AST, aspartate aminotransferase; ALT, alanine aminotransferase; GT, γ-glutamyl transpeptidase; ALP, alkaline phosphatase; CLT, cholinesterase; TBC, total bile acid; GLC, glucose; UAM, urea; UAC, uric acid; CRE, creatinine; SHP, serum hydrogen phosphate; K, potassium; Na, sodium; CL, chlorine; Ca, calcium; P, phosphorus; Mg, magnesium; CYS, homocysteine; CK, creatine kinase; WBC, white blood cell count; RBC, red blood cell count; PLT, platelet count.

Meanwhile, a correlation heat map based on the Pearson’s correlation coefficients was used to examine the relationship among these blood traits (Figure 1). Based on the analysis results, moderate to strong correlations (|r| > 0.6) among blood traits were identified. These correlation patterns were used to describe the relationships among traits; all subsequent association analyses were performed using univariate GWAS, as described in the Methods. The analysis revealed that several traits showed negative correlations with other traits, including CK, AST, ALT, GT, and ALP; although these correlations did not reach statistical significance, their direction was consistently negative. In contrast, certain electrolytes, such as K, Na, Cl, and Ca, showed weak positive correlations with other parameters, particularly with the hematological markers WBC, RBC, and PLT. The Mantel test revealed no significant overall correlation between the hematological and biochemical parameter matrices (Mantel’s r = 0.1354, *p* = 0.132). Spearman correlation coefficients among the 30 blood parameters are provided in Table S2; the results were broadly consistent with the Pearson correlation matrix.

### 3.2. Signals of Selection on Candidate Genes in Yunling Cattle

**Figure 2:**
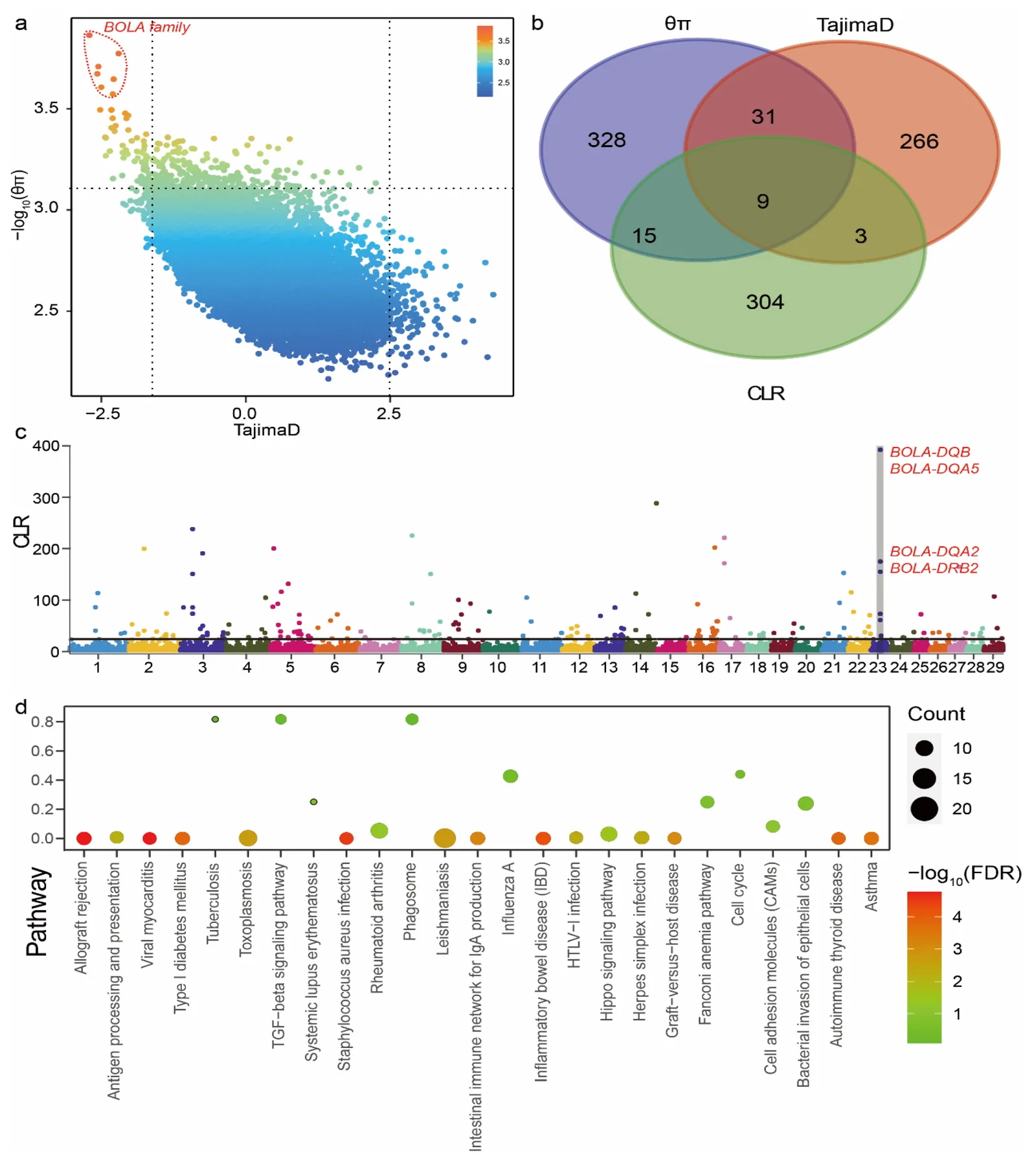
Scanning analysis of genomic selection signals in Yunling cattle. (**a**) Scatter plot of nucleotide diversity and Tajima’s D analysis. (**b**) Venn diagram of the selected region genes from three methods. (**c**) Manhattan plot of CLR results. (**d**) Gene enrichment analysis of the candidate genes.

To identify selective signatures, we calculated nucleotide diversity (θπ), Tajima’s D and the Composite Likelihood Ratio (CLR) in Yunling cattle (Figure 2a). The significant selective signal results were presented in Table S3. A total of 383, 309 and 331 genes were annotated for the three methods, respectively (Figure 2b). Regions with overlapping outlier signals between two or more methods were considered as candidate regions under selection. Among these candidate regions, nine genes (*CYM*, *PROK1*, *SPATA1*, *GNG5*, *CTBS*, *REV3L*, *SLC16A10*, *BOLA-DQB* and *BOLA-DQA5*) appeared in the overlap of all three methods. *BOLA-DQB* and *BOLA-DQA5* on BTA 23 were located in the most significant region of the three methods, while, *BOLA-DQA2* and *BOLA-DRB2* were also located in the selected region of CLR (Figure 2c). Kyoto Encyclopedia of Genes and Genomes (KEGG) and Gene Ontology (GO) enrichment analysis of candidate genes was performed. A large number of immunity disease resistance and antibody production-related signaling pathways (allograft rejection, graft-versus-host disease, intestinal immune network for IgA production, autoimmune thyroid disease, viral myocarditis, and antigen processing and presentation) were significantly enriched (FDR < 0.05). Moreover, *BOLA-DQB*, *BOLA-DQA5*, *BOLA-DQA2* and *BOLA-DRB2* which belong to the major histocompatibility complex (MHC) gene family were involved in three immune-related KEGG pathways (intestinal immune network for IgA production, antigen processing and presentation, and autoimmune thyroid disease) (Figure 2d, Table S4).

### 3.3. Genome-Wide Association Analysis of Hematological and Biochemical Traits

The GWAS results of the blood traits were presented in Supplementary Tables S5–S11. The QQ plots showed that the observed *p* values closely followed the expected null distribution, with deviation only in the upper tail corresponding to the significant associations (Supplementary Figure S1). In total, 623 suggestive SNPs (*P*_wald_ < 1 × 10^−6^) and 35 significant SNPs (*P*_wald_ < 5 × 10^−8^) were detected for 22 biochemical traits and 8 haematological traits (30 traits in total). For each significant locus, the distance between the lead SNP and the nearest annotated gene is reported in Supplementary Tables S3–S9. Where the associated variant is located within a gene (e.g., intronic or exonic), the gene name is given directly; where the variant lies in an intergenic region, the distance to the nearest gene is indicated. Among these traits, only 7 traits (ALB, CYS, WBC, BIL, LD-CHO, RDW and I-BIL) contained significant SNPs.

**Figure 3:**
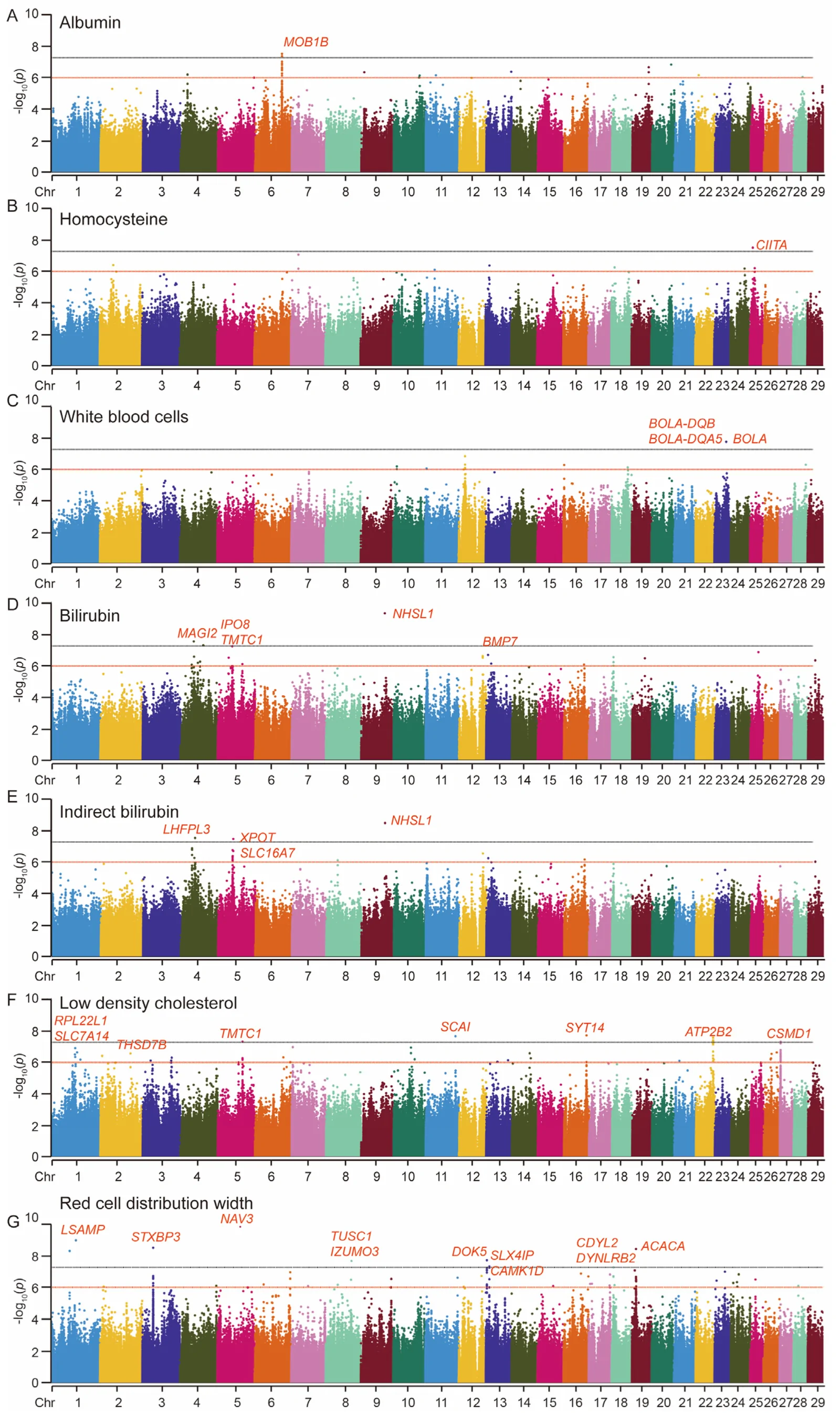
Manhattan plot for GWAS analysis of 7 hematological and biochemical traits. (**A**) Albumin trait. (**B**) Homocysteine trait. (**C**) White blood cells trait. (**D**) Bilirubin trait. (**E**) Indirect bilirubin trait. (**F**) Low-density cholesterol trait. (**G**) Red cell distribution width trait. The red and grey line indicate the significant threshold and suggestive threshold, respectively.

Albumin (ALB), synthesized in the liver of animals, exerts a significant influence on various antibodies and trace elements within the circulatory system. In addition to its role as a plasma expander, albumin plays pivotal roles in immune modulation, as well as the binding and transportation of both endogenous and exogenous substances. Through comprehensive genomic association analysis of albumin, 20 suggestive and 3 significantly associated loci were identified. Further annotation revealed that three of these loci were situated on chromosome 6 within the *MOB1B* gene (Figure 3A, Table S5).

Homocysteine (CYS) is a sulfur-containing amino acid and an important metabolic biomarker. The genome-wide association scan for Homocysteine revealed 15 suggestive and 1 significantly associated loci with annotation depicting the prevalence of the significant loci in the *CIITA* gene on chromosome 25 (Figure 3B, Table S6).

Blood cells are essential components of blood and are primarily classified into white blood cells (WBC), red blood cells (RBC), and platelets (PLT). WBCs include neutrophils, lymphocytes, monocytes, eosinophils, and basophils. As a key part of the immune system, WBCs play a vital role in protecting the body against diseases and harmful substances from the external environment by participating in immune responses. In the current study, a genome-wide association study (GWAS) was conducted on white blood cell traits, identifying 18 suggestive and 3 genome-wide significant loci. These three significant loci were located within the *BOLA*, *BOLA-DQB*, and *BOLA-DQA5* genes (Figure 3C, Table S7).

Bilirubin (BIL) is a potent antioxidant that is commonly associated with liver-related organ diseases in animals. GWAS was performed on total serum bilirubin levels in Yunling cattle. A total of 31 suggestive and 3 genome-wide significant loci were identified. Annotation of these significant loci revealed that the most significant locus was located within the *NHSL1* gene on chromosome 9 (Figure 3D). Another significant locus was found on chromosome 5, situated near the *IPO8* (distance = 872,483 bp) and *TMTC1* (distance = 13,027 bp) genes. The third significant locus was annotated to the *MGI2* gene on chromosome 4. Additionally, among the suggestive loci, *BMP7*, a member of the bone morphogenetic protein family, which is known to be involved in skeletal development, was identified (Table S8).

Indirect bilirubin (I-BIL), another key indicator of blood physiological and biochemical status, was also analyzed through GWAS. A total of 30 suggestive loci were identified along with 4 genome-wide significant loci. These loci exhibited a distribution pattern similar to that of total bilirubin, predominantly clustering on chromosomes 4, 5, and 9. However, the associated genes differed in part. The primary genes associated with I-BIL included *LHFPL3* on chromosome 4, *XPOT*, and *SLC16A7* (distance = 634,478 bp) on chromosome 5. Notably, consistent with total bilirubin, the most significant locus for I-BIL was also located within the *NHSL1* gene on chromosome 9 (Figure 3E, Table S9).

Blood cholesterol concentration is closely linked to cardiovascular diseases. Recent GWAS across various animal species have identified numerous candidate loci associated with the expression levels of total cholesterol (CHO), high-density lipoprotein cholesterol (HD-CHO), and low-density lipoprotein cholesterol (LD-CHO). In the present study, a GWAS of LD-CHO revealed 82 suggestive and 8 genome-wide significant loci. Annotation of the significant loci showed that the most significant locus was located on chromosome 1 and was associated with the *RPL22L1* and *SLC7A14* genes. The remaining significant loci were annotated to *THSD7B* on chromosome 2, *TMTC1* on chromosome 5, *SCAI* on chromosome 11, *SYT14* on chromosome 16, *ATP2B2* on chromosome 22, and *CSMD1* on chromosome 27 (Figure 3F, Table S10).

Typically, the analysis of red blood cell–related parameters in blood includes four key indicators: red blood cell count (RBC), hematocrit (HCT), mean corpuscular volume (MCV), and red cell distribution width (RDW). A large number of significant signals were identified in the GWAS for RDW, comprising 65 suggestive loci and 13 genome-wide significant loci. The most significant locus was found in the *NAV3* gene on chromosome 5. Moreover, two significant loci were detected on chromosome 1, primarily associated with the *LSAMP* gene. In addition, one significant locus was located on chromosome 3 within the *STXBP3* gene. A significant locus on chromosome 8 was identified near the *TUSC1* and *IZUMO3* genes. Furthermore, chromosome 13 harbored four significant loci involving the *DOK5*, *SLX4IP*, and *CAMK1D* genes, while chromosome 18 revealed three significant loci associated with *CDYL2* and *DYNLRB2*. Lastly, another significant locus located on chromosome 19 was found associated with the *ACACA* gene (Figure 3G, Table S11).

Integration of GWAS and selection signals. The most notable overlap between GWAS and selection signals was observed for the *BOLA* gene family. The three significant loci associated with white blood cell count (WBC) were located within *BOLA*, *BOLA-DQB*, and *BOLA-DQA5* on chromosome 23. Notably, *BOLA-DQB* and *BOLA-DQA5* were also identified as overlapping outlier genes by all three selection-signature methods (θπ, Tajima’s D, and CLR) on BTA23, and were located within the most significant selection region detected by these methods. This overlap suggests that the *BOLA* gene family may have been a target of historical selection in Yunling cattle and may contribute to the breed’s adaptive immune advantages.

## 4. Discussion

Blood physiological and biochemical indicators are widely recognized as key biomarkers for disease diagnosis in clinical practice. Studies have shown that the genetic contribution to these indicators varies considerably: some, such as the electrolytes sodium, calcium, and chloride, are largely acquired and can be modulated by external factors such as diet and management [22,23], whereas most other blood physiological and biochemical indicators are predominantly determined by genetic factors [24,25,26]. This provides us with an opportunity to uncover the genetic basis underlying these blood physiological and biochemical indicators at the whole-genome level. Currently, there are numerous studies on human blood biochemical indicators, and a substantial body of literature has also addressed blood parameters in pigs, where genome-wide association studies have identified numerous QTLs and candidate genes for hematological and biochemical traits [27,28,29]. In cattle, however, research on the genetic architecture of blood parameters remains relatively limited, with most studies focusing on Holstein dairy cattle [30]. Studies on indigenous Chinese cattle breeds are particularly scarce. Blood physiological and biochemical indicators can not only reflect the clinical disease indicators of livestock and poultry but also reflect the body’s metabolic indicators.

In this study, we performed a genome-wide association analysis on multiple quantitative traits of blood physiological and biochemical indicators in Yunling cattle; we identified seven blood physiological and biochemical indicator traits with 35 significant associated SNPs. The seven traits can be classified into functional groups based on their functions and components: lipid-related parameters (low-density lipoprotein), metabolic and protein-related parameters (bilirubin, indirect bilirubin, and albumin), homocysteine-related parameters (Homocysteine), white blood cell-related parameters (white blood cell count), and red blood cell-related parameters (red blood cell distribution width). The 35 significant loci associated with the traits mainly involve 22 genes (*MOB1B*, *CIITA*, *BOLA*, *BOLA-DQB*, *BOLA-DQA5*, *IPO8*, *TMTC1*, *LHFPL3*, *NHSL1*, *RPL22L1*, *SLC7A14*, *XPOT*, *SLC16A7*, *ATP2B2*, *TUSC1*, *IZUMO3*, *DOK5*, *SLX4IP*, *CAMK1D*, *CDYL2*, *DYNLRB2* and *ACACA*). Genes at distances greater than 100 kb from the associated variant (including *SLC16A7*, *IPO8*, *TUSC1* and *DYNLRB2*) are described as positional candidates only. We acknowledge that with 97 animals, the present study has limited statistical power and can only detect variants of relatively large effect; the effect sizes of the significant variants are therefore likely to be upwardly biased. All identified loci should be regarded as candidate associations requiring validation in larger and independent populations.

The *BOLA* gene family and KEGG enrichment. The three significant SNPs associated with white blood cell count were located within the *BOLA*, *BOLA-DQB*, and *BOLA-DQA5* genes on chromosome 23. These genes belong to the bovine major histocompatibility complex (MHC) and play a central role in antigen presentation and immune response. Notably, *BOLA-DQB* and *BOLA-DQA5* were also identified as overlapping outlier genes by all three selection-signature methods on BTA23, suggesting that this region may have been a target of historical selection in Yunling cattle. However, we acknowledge that the enrichment of immune-related pathways is largely driven by the four paralogous *BOLA* genes (*BOLA-DQB*, *BOLA-DQA5*, *BOLA-DQA2*, and *BOLA-DRB2*) within the MHC region, rather than by multiple independent loci. Therefore, the enrichment of these pathways should be interpreted with caution.

The *MOB1B* gene is a core component of the Hippo signaling pathway, which regulates cell proliferation and apoptosis to control the development and normal function of biological organs [31]. The current study found a significant correlation between this gene and the circulating blood albumin concentration (ALB) in Yunling cattle. Albumin is the most abundant plasma protein synthesized in the liver, and is essential for maintaining the osmotic pressure and transporting a wide range of endogenous and exogenous compounds within the cell. These findings suggest that *MOB1B* may be involved in regulating blood albumin content in Yunling cattle; however, functional validation is required.

Homocysteine is a sulfur-containing amino acid and is increasingly considered an important metabolic biomarker. The *CIITA* gene was significantly associated with homocysteine levels in Yunling cattle. *CIITA* is a transcriptional activator specific to MHC class II genes and plays an important role in immune regulation [32,33,34,35]. Given that Yunling cattle are known to exhibit tick resistance, the association between *CIITA* and homocysteine levels observed here may be relevant to immune-related traits. However, no tick burden or immune challenge was measured in this study, and this hypothesis requires further investigation.

Bilirubin and indirect bilirubin are widely used indicators of liver function and metabolism. In this study, *NHSL1* was significantly associated with both bilirubin and indirect bilirubin. *NHSL1* has previously been reported to be associated with primary hypertension in humans [36], and bilirubin levels have been reported to be inversely associated with hypertension in a Chinese ageing cohort [37,38]. Furthermore, Coltell et al. (2019) conducted a genome-wide association analysis of blood bilirubin levels in populations with different metabolic capacities, analyzing the key candidate genes that affect blood bilirubin levels and the relationship between body bilirubin levels, dietary habits, and gender differences [39]. In this study, the genes *IPO8*, *TMTC1*, *MGI2*, *LHFPL3*, *XPOT*, *SLC16A7*, and *NHSL1* were significantly associated with bilirubin and indirect bilirubin. For genes at large distances, particularly *IPO8* and *SLC16A7*, we describe them as positional candidates only. *SLC16A7*, a monocarboxylate transporter (MCT2), has been associated with growth, fat deposition, meat quality, and metabolic traits. Fonseca et al. (2020) found that *SLC16A7* has a certain genetic effect in regulating subcutaneous fat and muscle fat formation through gene expression profiling and a genome-wide association analysis of Nellore and Wagyu beef [40]. Abousoliman et al. (2021) conducted a genome-wide association analysis of growth traits in Egyptian sheep and found that *SLC16A7* is significantly associated with body weight, hip height, and chest girth [41]. *SLC16A7* is located 634,478 bp from the associated variant; therefore, this gene should be regarded as a positional candidate only. Further functional evidence is required before any role in regulating bilirubin levels can be proposed.

For LD-CHO, the strongest association signal was located on chromosome 1 near *RPL22L1* and *SLC7A14*. *RPL22L1* encodes a ribosomal protein, and while its role in lipid metabolism has not been established, it represents a positional candidate for further investigation. SLC7A14 is a cationic amino acid transporter; a recent study in mice showed that hypothalamic SLC7A14 indirectly influences lipolysis, suggesting a possible link to lipid metabolism that warrants validation in cattle [42]. *ATP2B2* encodes a plasma membrane calcium-transporting ATPase that regulates intracellular calcium homeostasis. Although *ATP2B2* is not directly involved in cholesterol metabolism, calcium signalling has been implicated in lipid metabolism and adipogenesis [43]. Therefore, the association between *ATP2B2* and LD-CHO observed here may reflect a role of calcium signalling in lipid regulation, but this hypothesis requires further investigation.

In summary, the current study identifies several candidate genes, including *MOB1B* (albumin), *CIITA* (Homocysteine), and *SLC16A7* (bilirubin). These loci should be regarded as candidate associations that provide a foundation for future functional studies. Larger datasets, replication cohorts, and validation studies are required to confirm their roles in Yunling cattle.

Generalizability to other breeds. The findings of this study are based on a single composite breed, Yunling cattle, which has a complex ancestry (approximately 56% Indian zebu, 40% European taurine, and 4% Chinese zebu). Given this composite ancestry, extrapolation to other indigenous Chinese breeds should be made with caution. Nevertheless, several of the candidate genes identified here are located in genomic regions that are conserved across cattle breeds, and some have been associated with blood traits in other breeds. For example, the *BOLA* genes are part of the major histocompatibility complex and are known to play a role in immune regulation in multiple cattle breeds. Similarly, *SLC16A7* has been associated with metabolic traits in other cattle populations. These observations suggest that some of the associations identified here may warrant investigation in other cattle populations; however, breed-specific genetic background, population history, and environmental factors may influence the strength and direction of these associations. Therefore, validation in other breeds, and in larger and more diverse populations, is required before any broader application of these findings can be recommended.

## 5. Conclusions

This study provides a genome-wide characterization of the genetic architecture of blood physiological and biochemical traits in Yunling cattle. Several candidate genes and regions were identified, primarily *MOB1B* (potentially associated with albumin levels) and *CIITA* (potentially associated with homocysteine levels). *SLC16A7,* located 634 kb from the lead SNP, is described as a positional candidate only. These findings expand the current knowledge of the genetic basis of blood traits in indigenous Chinese cattle, a group for which such data have been largely unavailable. Given the relatively small sample size (97 animals), all identified loci should be regarded as promising candidate markers rather than validated markers for breeding programmes. These candidate loci provide a foundation for further functional validation and for marker-assisted selection aimed at improving blood-related traits in indigenous cattle breeds. Validation in larger and independent populations, together with further functional and multi-omics studies, is needed before any application.

## Figures and Tables

**Table 1:** Descriptive statistics of hematological and biochemical traits.

Traits	Units	Minimum	Maximum	Mean	*SD*	CV (%)	Kurtosis	Skewness
TG	mmol/L	0.11	0.54	0.25	0.1	0.02	0.33	0.78
CHO	mmol/L	1.97	5.36	3.33	0.8	0.16	−0.37	0.53
HD-CHO	mmol/L	1.44	3.11	2.21	0.39	0.08	−0.36	0.11
LD-CHO	mmol/L	0.02	0.8	0.31	0.15	0.03	0.84	0.68
BIL	μmol/L	1	6.6	2.78	1.03	0.21	0.95	0.65
DBIL	μmol/L	0.3	2.1	1.12	0.37	0.08	−0.02	0.19
IBIL	μmol/L	0.4	4.5	1.66	0.79	0.16	0.79	0.84
PRO	g/L	47.9	95.4	81.04	7.84	1.58	2.03	−0.68
ALB	g/L	26.7	42.4	35.55	2.68	0.54	1.50	−0.78
GLO	g/L	21.2	67.5	45.49	8.08	1.63	−0.07	−0.01
PALB	mg/L	25	416	45.31	42.21	8.51	64.59	7.69
AST	U/L	35	195	87.56	32.48	6.55	1.23	1.19
ALT	U/L	12	50	28.11	7.6	1.53	0.26	0.48
GT	U/L	1	143	26.82	22.55	4.54	9.56	2.83
ALP	U/L	30	298	100.19	50.38	10.15	2.62	1.44
CLT	U/L	120	401	243.13	54.73	11.03	0	−0.03
TBC	μmol/L	3.9	90.4	20.12	14.78	2.98	5.09	1.88
GLC	mmol/L	2.54	9.35	4.26	1.02	0.21	10.33	2.66
Urea	mmol/L	2.16	8.67	4.97	1.64	0.33	−0.75	0.27
UAC	μmol/L	16	82	42.37	14.3	2.88	−0.4	0.29
CRE	μmol/L	72	253	122.06	35.72	7.2	2.01	1.35
SHP	mmol/L	14.4	32.3	25.82	3.03	0.61	1.97	−0.88
K	mmol/L	2.22	5.86	4.17	0.52	0.11	1.95	0.08
Na	mmol/L	133.1	149	141.49	3.16	0.64	−0.06	−0.4
CL	mmol/L	77.2	106.9	98.7	4.11	0.83	7.13	−1.86
Ca	mmol/L	1.99	2.63	2.36	0.12	0.02	0.06	−0.39
P	mmol/L	0.49	3.46	1.71	0.56	0.11	1.03	0.77
Mg	mmol/L	0.77	1.21	0.95	0.1	0.02	−0.49	0.1
CYS	μmol/L	0	13.6	6	2.88	0.58	−0.41	0.49
CK	U/L	54	1431	192.65	209.79	42.28	20.19	4.28
WBC	×10^9^/L	4.95	15.63	9.62	2.44	0.5	−0.5	0.44
RBC	×10^12^/L	5.18	10.82	8.22	0.91	0.19	1.17	0.2
HGB	g/L	112	194	147.26	14.01	2.89	1.22	0.24
HCT	L/L	0.31	0.5	0.4	0.04	0.01	0.02	0.22
MCV	fL	37	64.1	48.97	5.21	1.07	0.18	0.24
MCH	pg	14.3	21.8	17.99	1.38	0.28	−0.06	0.02
MCHC	g/L	315	401	368.75	16.99	3.5	0.18	−0.39
RDW	%	18	29	23.2	1.97	0.41	0.91	0.74
PLT	×10^9^/L	8.	487	262.51	98.9	20.37	−0.02	−0.19

Abbreviations: ALB, albumin; ALP, alkaline phosphatase; ALT, alanine aminotransferase; AST, aspartate aminotransferase; BIL, total bilirubin; Ca, calcium; CHO, total cholesterol; CK, creatine kinase; CL, chlorine; CLT, cholinesterase; CYS, homocysteine; DBIL, direct bilirubin; GLC, glucose; GLO, globulin; GT, γ-glutamyl transpeptidase; HCT, hematocrit; HD-CHO, high-density lipoprotein cholesterol; HGB, hemoglobin; IBIL, indirect bilirubin; K, potassium; LD-CHO, low-density lipoprotein cholesterol; MCH, mean corpuscular hemoglobin; MCHC, mean corpuscular hemoglobin concentration; MCV, mean corpuscular volume; Mg, magnesium; Na, sodium; P, phosphorus; PALB, prealbumin; PLT, platelet count; PRO, total proteins; RBC, red blood cell count; RDW, red cell distribution width; SHP, serum hydrogen phosphate; TG, triglycerides; UAC, uric acid; Urea, urea; WBC, white blood cell count.

## Data Availability

Sequences are available from GenBank with the Bioproject accession number PRJNA555741. The phenotype data and GWAS summary statistics generated in this study are available from the corresponding author upon reasonable request.

## Supplementary Materials

The following supporting information can be downloaded at https://www.mdpi.com/article/10.3390/ani16203302/s1.

## Abbreviations

The following abbreviations are used in this manuscript:

Chol	Cholesterol
HD-Chol	High-Density Lipoprotein Cholesterol
LD-Chol	Low-Density Lipoprotein Cholesterol
TG	Triglycerides
GLU	Glucose
UAM	Urea Ammonia
UAC	Uric Acid
T-Bil	Total Bilirubin
D-BIL	Direct Bilirubin
I-BIL	Indirect Bilirubin
T-Prot	Total Proteins
ALB	Albumin
GLO	Globulin
PAB	Prealbumin
ALP	Alkaline Phosphatase
ALT	Alanine Aminotransferase
AST	Aspartate Aminotransferase
GLT	Γ-Glutamyl Transpeptidase
CLT	Cholinesterase
CK	Creatine Kinase
SHP	Serum Hydrogen Phosphate
K	Potassium
Na	Sodium
CL	Chlorine
Ca	Calcium
P	Phosphorus
Mg	Magnesium
RBC	Red Blood Cell Count
HGB	Hemoglobin
HCT	Hematocrit
MCV	Mean Corpuscular Volume
MCH	Mean Corpuscular Hemoglobin
MCHC	Mean Corpuscular Hemoglobin Concentration
RDW	Red Cell Distribution Width
WBC	White Blood Cell Count
PLT	Platelet Count

## References

[1] Qu K.X., Huang B.Z., Yang G.R., He Z.X., Zhang Y.P., Zan L.S. (2012). Genetic diversity analysis of BMY cattle based on microsatellite DNA markers. Genetika.

[2] Chen Q., Zhan J., Shen J., Qu K., Hanif Q., Liu J., Zhang J., Chen N., Chen H., Huang B. (2020). Whole-genome resequencing reveals diversity, global and local ancestry proportions in yunling cattle. J. Anim. Breed. Genet..

[3] Zhao G., Yu M., Cui Q., Zhou X., Zhang J., Li H., Qu K., Wang G., Huang B. (2013). Association of bovine Toll-like receptor 4 with tick infestation rates and blood histamine concentration. Genet. Mol. Res..

[4] Kvržić Z. (2024). Blood and the Circulatory System. History of Blood Donation and Transfusion Medicine.

[5] Alimov N. (2023). Blood supply to the human body, vascular anatomy and blood components. West. Eur. J. Med. Med. Sci..

[6] Ramaiah S. (2007). A toxicologist guide to the diagnostic interpretation of hepatic biochemical parameters. Food Chem. Toxicol..

[7] Das B., Ellis M., Sahoo M. (2024). Veterinary diagnostics: Growth, trends, and impact. Evolving Landscape of Molecular Diagnostics.

[8] Perera T.R., Skerrett-Byrne D.A., Gibb Z., Nixon B., Swegen A. (2022). The future of biomarkers in veterinary medicine: Emerging approaches and associated challenges. Animals.

[9] Evans D.M. (2020). It’s Bloody Genes!. Twin Res. Hum. Genet..

[10] Shi K., Niu F., Zhang Q., Ning C., Yue S., Hu C., Xu Z., Wang S., Li R., Hou Q. (2020). Identification of whole-genome significant single nucleotide polymorphisms in candidate genes associated with serum biochemical traits in Chinese Holstein cattle. Front. Genet..

[11] Kim Y., Lee Y., Lee S., Kim N.H., Lim J., Kim Y.J., Oh J.H., Min H., Lee M., Seo H.-J. (2015). On the estimation of heritability with family-based and population-based samples. BioMed Res. Int..

[12] Yan Z., Huang H., Freebern E., Santos D.J., Dai D., Si J., Ma C., Cao J., Guo G., Liu G.E. (2020). Integrating RNA-Seq with GWAS reveals novel insights into the molecular mechanism underpinning ketosis in cattle. BMC Genom..

[13] Danecek P., Auton A., Abecasis G., Albers C.A., Banks E., DePristo M.A., Handsaker R.E., Lunter G., Marth G.T., Sherry S.T. (2011). The variant call format and VCFtools. Bioinformatics.

[14] DeGiorgio M., Huber C.D., Hubisz M.J., Hellmann I., Nielsen R. (2016). SweepFinder2: Increased sensitivity, robustness and flexibility. Bioinformatics.

[15] Chen Q., Huang B., Zhan J., Wang J., Qu K., Zhang F., Shen J., Jia P., Ning Q., Zhang J. (2020). Whole-genome analyses identify loci and selective signals associated with body size in cattle. J. Anim. Sci..

[16] Yang Y., Yang S., Tang J., Ren G., Shen J., Huang B., Lei C., Chen H., Qu K. (2022). Comparisons of hematological and biochemical profiles in Brahman and Yunling cattle. Animals.

[17] Kai W., Mingyao L., Hakon H. (2010). ANNOVAR: Functional annotation of genetic variants from high-throughput sequencing data. Nucleic Acids Res..

[18] Dechao B., Haitao L., Peipei H., Zhihao W., Shan Z., Zihao H., Yang W., Lianhe Z., Jingjia L., Jincheng G. (2021). KOBAS-i: Intelligent prioritization and exploratory visualization of biological functions for gene enrichment analysis. Nucleic Acids Res..

[19] Zhou X., Stephens M. (2012). Genome-wide efficient mixed-model analysis for association studies. Nat. Genet..

[20] Shen J.-F., Chen Q.-M., Zhang F.-W., Hanif Q., Huang B.-Z., Chen N.-B., Qu K.-X., Zhan J.-X., Chen H., Jiang Y. (2022). Genome-wide association study identifies quantitative trait loci affecting cattle temperament. Zool. Res..

[21] Anderson C.A., Pettersson F.H., Clarke G.M., Cardon L.R., Morris A.P., Zondervan K.T. (2010). Data quality control in genetic case-control association studies. Nat. Protoc..

[22] Whitfield J.B., Martin N.G. (1984). The Effects of Inheritance on Constituents of Plasma: A Twin Study on Some Biochemical Variables. Ann. Clin. Biochem..

[23] Simon M., Bod’A K., Greksák M., Kubinec J., Abrahámová D., Cerná S. (1978). Genetically conditioned variability of metabolic profile parameters in dairy cows. Veterinární Medicína.

[24] Lin J.-P., Zheng G., Joo J., Cupples L.A. (2010). Genome-wide linkage and association scans for quantitative trait loci of serum lactate dehydrogenase—The framingham heart study. Hum. Genom. Proteom..

[25] Srivastava A., Masinde G., Yu H., Baylink D., Mohan S. (2004). Mapping quantitative trait loci that influence blood levels of alkaline phosphatase in MRL/MpJ and SJL/J mice. Bone.

[26] Baes C., Plastow G., Colazo M., Liu R., Miglior F., Hailemariam D., Stothard P. (2025). Genetic parameters and QTL mapping for novel metabolic traits in early-lactation Holsteins. J. Anim. Sci..

[27] Bovo S., Mazzoni G., Bertolini F., Schiavo G., Galimberti G., Gallo M., Dall’Olio S., Fontanesi L. (2019). Genome-wide association studies for 30 haematological and blood clinical-biochemical traits in Large White pigs reveal genomic regions affecting intermediate phenotypes. Sci. Rep..

[28] Luo W., Chen S., Cheng D., Wang L., Li Y., Ma X., Song X., Liu X., Li W., Liang J. (2012). Genome-wide Association Study of Porcine Hematological Parameters in a Large White × Minzhu F2 Resource Population. Int. J. Biol. Sci..

[29] Ponsuksili S., Reyer H., Trakooljul N., Murani E., Wimmers K. (2016). Single- and Bayesian Multi-Marker Genome-Wide Association for Haematological Parameters in Pigs. PLoS ONE.

[30] Yang T., Luo H., Lou W., Chang Y., Brito L.F., Zhang H., Ma L., Hu L., Wang A., Li S. (2024). Genetic background of hematological parameters in Holstein cattle based on genome-wide association and RNA sequencing analyses. J. Dairy Sci..

[31] Meng Z., Moroishi T., Guan K.-L. (2016). Mechanisms of Hippo pathway regulation. Genes Dev..

[32] Machado J.A.L., Steimle V. (2021). The MHC class II transactivator CIITA: Not (quite) the odd-one-out anymore among NLR proteins. Int. J. Mol. Sci..

[33] Hou J., Chen S.N., Gan Z., Li N., Huang L., Huo H.J., Yang Y.C., Lu Y., Yin Z., Nie P. (2020). In primitive zebrafish, MHC class II expression is regulated by IFN-γ, IRF1, and two forms of CIITA. J. Immunol..

[34] Liu C., Ruan P., Wang Z., Huang Y., Zhang L., Yu D., Huang D., Ceccobelli S., Gao H., E G. (2026). Multi-omics dissection of heat stress reveals CIITA as a central regulator of metabolic thermotolerance in cattle (*Bos taurus*). Commun. Biol..

[35] Sylvester H., Koganti P.P., Gurung S., Pillai V.V., Selvaraj V. (2025). From blood to pluripotency: Fibrocytes as a reprogrammable somatic cell source for bovine iPSCs. bioRxiv.

[36] Dai X., Wu C., He Y., Gui L., Zhou L., Guo H., Yuan J., Yang B., Li J., Deng Q. (2014). A Genome-Wide Association Study for Serum Bilirubin Levels and Gene-Environment Interaction in a Chinese Population. Genet. Epidemiol..

[37] Fowdar J.Y., Grealy R., Lu Y., Griffiths L.R. (2017). A genome-wide association study of essential hypertension in an Australian population using a DNA pooling approach. Mol. Genet. Genom..

[38] Tang C., Jiang H., Zhao B., Lin Y., Lin S., Chen T., Su Y., Zhang Y., Zhou L., Li L. (2022). The association between bilirubin and hypertension among a Chinese ageing cohort: A prospective follow-up study. J. Transl. Med..

[39] Coltell O., Asensio E.M., Sorlí J.V., Barragán R., Fernández-Carrión R., Portolés O., Ortega-Azorín C., Martínez-LaCruz R., González J.I., Zanón-Moreno V. (2019). Genome-Wide Association Study (GWAS) on bilirubin concentrations in subjects with metabolic syndrome: Sex-specific GWAS analysis and gene-diet interactions in a Mediterranean population. Nutrients.

[40] Fonseca L.F.S., Dos Santos Silva D.B., Gimenez D.F.J., Baldi F., Ferro J.A., Chardulo L.A.L., de Albuquerque L.G. (2020). Gene expression profiling and identification of hub genes in Nellore cattle with different marbling score levels. Genomics.

[41] Abousoliman I., Reyer H., Oster M., Murani E., Mohamed I., Wimmers K. (2021). Genome-Wide Analysis for Early Growth-Related Traits of the Locally Adapted Egyptian Barki Sheep. Genes.

[42] Jiang X., Liu K., Luo P., Li Z., Xiao F., Jiang H., Wu S., Tang M., Yuan F., Li X. (2024). Hypothalamic SLC7A14 accounts for aging-reduced lipolysis in white adipose tissue of male mice. Nat. Commun..

[43] Xu L., Wang Z., Xiong X., Gu X., Gao X., Gao X. (2011). Identification of a novel point mutation of mouse Atp2b2 induced by N-ethyl-N-nitrosourea mutagenesis. Exp. Anim..

