# Supplementary material for: Genome-Wide Association and Selection Signature Analyses of Hematological and Serum Biochemical Traits in Yunling Cattle
Source: Animals (Basel). 2026 Oct 20;16(20):3302. doi: 10.3390/ani16203302 (PMC13700815; doi:10.3390/ani16203302)
Supplement: Supplementary file 1 [file animals-16-03302-s001.zip › animals-4551155-supplementary.pdf]

Table S1 Summary statistics of whole-genome resequencing data for the 97 Yunling cattle

Sample ID	Accession number	Number of clean reads	Number of Bases	Average length of reads	Ratio of high-quality reads (>Q20)	Mapping rate	Mean depth (X)
CDY006	SRR9721837	95512551	14019305607	146.78	0.9996	0.9964	6.2993
CDY016	SRR9721838	112046683	16532605938	147.55	0.9997	0.9944	5.5567
CDY023	SRR9721839	105495939	15563230223	147.52	0.9997	0.9966	5.7039
CDY026	SRR9721832	123469886	18223913994	147.6	0.9997	0.9956	5.0891
CDY028	SRR9721833	104672882	15418531191	147.3	0.9997	0.9968	5.7961
CDY029	SRR9721834	114813218	16920625427	147.38	0.9997	0.997	5.1352
CDY031	SRR9721835	111591065	16223389860	145.38	0.9995	0.9971	5.6421
CDY035	SRR9721840	106471835	15696585896	147.42	0.9996	0.9969	6.0833
CDY041	SRR9721841	79703938	11653149596	146.21	0.9995	0.9962	5.9431
CDY044	SRR9721763	90588062	13326180728	147.11	0.9996	0.9948	6.7862
CDY050	SRR9721762	105966369	15429482417	145.61	0.9995	0.996	5.7578
CDY052	SRR9721765	97207027	14270085675	146.8	0.9996	0.9967	5.4443
CDY055	SRR9721764	97570005	14350802989	147.08	0.9995	0.9953	5.7024
CDY056	SRR9721759	116299218	17070257106	146.78	0.9996	0.9953	4.4747
CDY058	SRR9721758	96325508	14172595795	147.13	0.9995	0.9961	5.8528
CDY061	SRR9721761	98758988	14510052500	146.92	0.9994	0.996	6.004
CDY069	SRR9721760	100703574	14748639243	146.46	0.9997	0.9953	5.1323
CDY075	SRR9721757	114286998	16777335918	146.8	0.9996	0.996	5.1562
CDY077	SRR9721756	107158419	15743827191	146.92	0.9996	0.9958	4.4264
CDY081	SRR9721785	107118007	15767835108	147.2	0.9996	0.995	5.8821
CDY086	SRR9721786	110548973	16275729161	147.23	0.9997	0.996	6.2296
CDY087	SRR9721783	117356806	17119386625	145.87	0.9995	0.9963	5.0668

CDY088	SRR9721784	108263905	15950697817	147.33	0.9996	0.996	5.0804
CDY089	SRR9721781	111973416	16533510686	147.66	0.9997	0.9963	4.984
CDY090	SRR9721782	93314452	13697643007	146.79	0.9994	0.9966	5.8351
CDY093	SRR9721779	127087375	18742826730	147.48	0.9995	0.996	5.9667
CDY106	SRR9721780	86809768	12772398228	147.13	0.9997	0.9946	4.9381
CDY110	SRR9721777	106957571	15787699116	147.61	0.9997	0.9953	6.5855
CDY132	SRR9721778	106730697	15726727665	147.35	0.9996	0.9959	5.317
CDY138	SRR9721855	113760864	16773300849	147.44	0.9997	0.995	5.5581
CDY161	SRR9721852	84050704	12338421166	146.8	0.9996	0.9965	6.3959
CDY195	SRR9721689	93070624	13615233645	146.29	0.9996	0.9955	5.8469
CDY201	SRR9721809	99890374	14660682227	146.77	0.9996	0.9952	5.7554
CDY204	SRR9721812	119714436	17636281042	147.32	0.9997	0.9957	5.5606
CDY206	SRR9721796	109888810	16123055397	146.72	0.9996	0.9954	6.0717
CDY207	SRR9721736	108110638	15869936472	146.79	0.9994	0.9959	4.8916
CDY210	SRR9721776	104277838	15332795978	147.04	0.9995	0.9967	6.1999
CDY216	SRR9721731	113743957	16742835816	147.2	0.9997	0.9957	5.2669
CDY217	SRR9721730	91976106	13488340980	146.65	0.9995	0.9954	5.048
CDY219	SRR9721749	115903905	17095377273	147.5	0.9997	0.9959	5.5163
CDY220	SRR9721750	98532360	14523232607	147.4	0.9997	0.9963	5.1856
CDY224	SRR9721751	94764564	13919734513	146.89	0.9995	0.9947	5.3894
CDY227	SRR9721752	103075016	15210342109	147.57	0.9997	0.9942	5.5627
CDY228	SRR9721754	97071261	14298601573	147.3	0.9997	0.9948	4.918
CDY234	SRR9721755	101242462	14860486154	146.78	0.9996	0.9967	4.7379
CDY235	SRR9721787	104331258	15338217787	147.01	0.9997	0.9966	6.4951
CDY240	SRR9721821	92229217	13561698668	147.04	0.9995	0.9957	5.6993
CDY241	SRR9721766	88473296	13064754755	147.67	0.9997	0.994	6.0691

CDY245	SRR9721768	121378879	17908587869	147.54	0.9998	0.9955	6.0064
CDY248	SRR9721767	107010173	15714866549	146.85	0.9997	0.9905	7.5329
CDY249	SRR9721753	113753033	16735288332	147.12	0.9997	0.9963	5.516
CDY250	SRR9721733	112571246	16560755771	147.11	0.9997	0.9952	5.4907
CDY251	SRR9721732	141817643	20770690005	146.46	0.9997	0.9961	5.9116
CDY252	SRR9721735	103292432	15209259227	147.24	0.9997	0.9947	6.5276
CDY255	SRR9721734	103046214	15139581678	146.92	0.9995	0.9951	5.0058
CDY256	SRR9721738	110525774	16300822312	147.48	0.9997	0.996	5.1465
CDY257	SRR9721737	122455128	18000129983	146.99	0.9995	0.9956	5.4814
CDY261	SRR9721719	93836269	13803085289	147.1	0.9997	0.9953	4.6534
CDY263	SRR9721718	96164332	14190938263	147.57	0.9997	0.9943	4.8896
CDY264	SRR9721712	102666102	15115333659	147.23	0.9996	0.9961	5.9464
CDY267	SRR9721713	87283882	12831618815	147.01	0.9996	0.9951	5.4542
CDY270	SRR9721710	91894445	13482819984	146.72	0.9995	0.9939	6.4698
CDY272	SRR9721711	111221310	16397570109	147.43	0.9996	0.9944	6.2333
CDY274	SRR9721690	102178384	15039218518	147.19	0.9997	0.9956	5.2247
CDY279	SRR9721691	122008784	17839591539	146.22	0.9994	0.9951	6.363
CDY280	SRR9721714	117367993	17187343665	146.44	0.9994	0.9958	5.063
CDY281	SRR9721715	97794824	14408567870	147.33	0.9997	0.9939	6.0927
CDY284	SRR9721853	119286919	17545535311	147.09	0.9994	0.9957	5.4675
CDY286	SRR9721854	94852958	13960698745	147.18	0.9997	0.9943	6.1355
CDY309	SRR9721740	115037729	16919318781	147.08	0.9996	0.9953	4.8609
CDY310	SRR9721739	91206193	13403273106	146.96	0.9996	0.994	5.9125
CDY315	SRR9721742	111159147	16302567263	146.66	0.9994	0.9962	4.8963
CDY320	SRR9721741	91831939	13501506185	147.02	0.9996	0.9947	6.4621
CDY323	SRR9721744	121452174	17818738354	146.71	0.9996	0.995	5.6905

CDY326	SRR9721743	106857928	15690652026	146.84	0.9995	0.9949	6.0009
CDY328	SRR9721746	112495252	16547515773	147.1	0.9995	0.9946	5.8944
CDY333	SRR9721745	110787021	16253359729	146.71	0.9996	0.9958	5.2307
CDY334	SRR9721748	98179358	14423352100	146.91	0.9995	0.9948	5.4921
CDY335	SRR9721747	103071110	15144230690	146.93	0.9995	0.9941	5.5754
CDY337	SRR9721722	104774344	15373502175	146.73	0.9995	0.9931	5.16
CDY338	SRR9721723	97206483	14228127098	146.37	0.9994	0.9954	5.9348
CDY339	SRR9721720	111553318	16363536991	146.69	0.9995	0.988	5.4601
CDY344	SRR9721721	99185301	14576978203	146.97	0.9995	0.9946	5.2864
CDY351	SRR9721726	113731324	16617856248	146.12	0.9995	0.9923	6.0265
CDY353	SRR9721727	112627642	16421188840	145.8	0.9994	0.9919	5.9551
CDY357	SRR9721724	108112874	15892341351	147	0.9996	0.9956	5.7636
CDY358	SRR9721725	90190423	13284809277	147.3	0.9997	0.9965	4.8176
CDY359	SRR9721728	121015036	17780123838	146.92	0.9996	0.9956	6.448
CDY365	SRR9721729	98837020	14449267866	146.19	0.9995	0.9956	5.2402
CDY367	SRR9721709	107827654	15683486537	145.45	0.9994	0.9888	5.6876
CDY369	SRR9721708	111873623	16529144033	147.75	0.9996	0.9971	5.9939
CDY371	SRR9721707	112433450	16449548144	146.3	0.9995	0.9953	5.9655
CDY374	SRR9721706	91708757	13534771461	147.58	0.9996	0.9968	4.9087
CDY375	SRR9721705	98251213	14510564890	147.69	0.9995	0.9964	5.2616
CDY380	SRR9721704	105161019	15465006509	147.06	0.9996	0.9962	5.6081
CDY387	SRR9721703	102830597	15080479868	146.65	0.9996	0.9958	5.4689
CDY388	SRR9721702	99750749	14716703918	147.53	0.9996	0.9966	5.337

Table S2. Spearman correlation coefficients among the 30 blood parameters retained after quality control in Yunling cattle

Traits	TG	CHO	HD-CHO	LD-CHO	BIL	DBIL	IBIL	PRO	ALB	GLO	AST	ALT	GT	ALP	CLT	TBC	GLC	UAM	UAC	CRE	SHP	K	Na	CL	Ca	P	Mg	CYS	CK	WBC	RBC	PLT
TG	1.00	-0.38	-0.26	-0.03	-0.31	-0.16	-0.29	-0.16	-0.05	-0.14	-0.29	-0.29	0.07	0.09	-0.07	0.12	0.20	-0.27	-0.23	0.28	0.06	0.09	0.23	0.19	0.14	0.06	0.29	0.43	-0.13	0.47	-0.01	-0.10
CHO	-0.38	1.00	0.71	0.21	0.26	0.08	0.33	0.21	0.38	0.13	0.29	0.27	0.21	0.16	0.23	0.26	-0.17	0.42	0.31	-0.06	0.06	0.17	-0.23	-0.04	0.26	-0.07	0.13	-0.44	-0.07	-0.23	0.04	0.02
HD-CHO	-0.26	0.71	1.00	0.01	0.24	0.02	0.32	0.42	0.15	0.38	0.31	0.22	0.30	0.08	0.41	0.36	-0.27	0.51	0.35	-0.31	0.20	-0.03	-0.34	-0.23	0.13	-0.39	-0.10	-0.44	-0.03	-0.22	-0.14	0.10
LD-CHO	-0.03	0.21	0.01	1.00	-0.29	-0.11	-0.25	-0.15	-0.17	-0.02	0.11	0.02	0.42	0.17	-0.17	-0.05	-0.17	-0.22	0.12	0.09	0.10	0.23	0.22	0.21	-0.06	0.47	0.33	0.17	0.11	0.20	-0.08	-0.13
BIL	-0.31	0.26	0.24	-0.29	1.00	0.53	0.94	0.39	0.39	0.26	0.19	0.10	-0.42	-0.29	0.49	0.00	0.19	0.42	0.09	-0.10	0.11	0.23	-0.10	-0.10	0.02	-0.40	-0.26	-0.36	0.05	-0.45	0.22	-0.17
DBIL	-0.16	0.08	0.02	-0.11	0.53	1.00	0.26	0.42	-0.14	0.41	-0.04	-0.06	-0.10	-0.20	0.16	-0.06	0.04	0.05	0.14	-0.15	-0.09	0.22	0.22	0.08	-0.12	-0.12	-0.12	-0.22	-0.26	-0.38	0.01	-0.06
IBIL	-0.29	0.33	0.32	-0.25	0.94	0.26	1.00	0.28	0.53	0.14	0.29	0.17	-0.40	-0.25	0.54	0.08	0.19	0.48	0.11	-0.06	0.17	0.21	-0.19	-0.16	0.12	-0.43	-0.24	-0.34	0.17	-0.35	0.26	-0.20
PRO	-0.16	0.21	0.42	-0.15	0.39	0.42	0.28	1.00	-0.24	0.96	0.10	-0.11	-0.07	-0.08	0.27	-0.03	-0.26	0.21	0.29	-0.52	0.12	-0.07	-0.07	-0.08	-0.05	-0.32	-0.42	-0.42	-0.23	-0.34	-0.21	0.18
ALB	-0.05	0.38	0.15	-0.17	0.39	-0.14	0.53	-0.24	1.00	-0.45	-0.07	0.18	-0.34	-0.15	0.33	0.10	0.24	0.40	-0.13	0.36	0.23	0.09	0.00	-0.05	0.48	-0.20	0.23	-0.04	0.06	0.00	0.25	-0.02
GLO	-0.14	0.13	0.38	-0.02	0.26	0.41	0.14	0.96	-0.45	1.00	0.11	-0.16	0.03	-0.04	0.17	-0.08	-0.32	0.11	0.32	-0.55	0.08	-0.09	-0.07	-0.03	-0.17	-0.25	-0.44	-0.36	-0.23	-0.30	-0.29	0.18
AST	-0.29	0.29	0.31	0.11	0.19	-0.04	0.29	0.10	-0.07	0.11	1.00	0.42	0.32	0.19	0.29	0.21	-0.31	0.46	0.58	-0.24	0.05	0.03	-0.31	-0.26	-0.08	-0.09	-0.26	-0.45	0.22	-0.07	0.12	-0.07
ALT	-0.29	0.27	0.22	0.02	0.10	-0.06	0.17	-0.11	0.18	-0.16	0.42	1.00	0.05	0.10	0.19	0.39	0.14	0.23	0.23	-0.08	-0.25	0.06	-0.15	-0.12	0.00	0.05	0.12	-0.36	0.55	-0.01	0.45	-0.12
GT	0.07	0.21	0.30	0.42	-0.42	-0.10	-0.40	-0.07	-0.34	0.03	0.32	0.05	1.00	0.48	-0.19	0.24	-0.29	0.04	0.33	-0.15	0.13	-0.19	-0.02	-0.11	0.07	0.23	0.22	-0.20	0.01	0.13	-0.20	-0.11
ALP	0.09	0.16	0.08	0.17	-0.29	-0.20	-0.25	-0.08	-0.15	-0.04	0.19	0.10	0.48	1.00	-0.05	0.15	-0.36	-0.09	0.33	-0.33	-0.13	-0.03	-0.01	0.17	0.08	0.25	0.38	0.11	0.34	-0.07	0.03	0.05
CLT	-0.07	0.23	0.41	-0.17	0.49	0.16	0.54	0.27	0.33	0.17	0.29	0.19	-0.19	-0.05	1.00	0.17	-0.18	0.53	0.18	-0.12	-0.15	0.16	-0.30	-0.13	-0.11	-0.56	-0.15	-0.28	0.03	-0.40	0.14	-0.11
TBC	0.12	0.26	0.36	-0.05	0.00	-0.06	0.08	-0.03	0.10	-0.08	0.21	0.39	0.24	0.15	0.17	1.00	0.10	0.12	0.14	-0.06	-0.25	0.11	-0.28	-0.28	0.11	-0.13	0.18	-0.04	0.26	0.07	0.24	-0.21
GLC	0.20	-0.17	-0.27	-0.17	0.19	0.04	0.19	-0.26	0.24	-0.32	-0.31	0.14	-0.29	-0.36	-0.18	0.10	1.00	-0.31	-0.61	0.34	-0.17	0.10	0.13	0.04	0.13	0.03	-0.02	0.14	0.34	0.25	0.36	-0.01
UAM	-0.27	0.42	0.51	-0.22	0.42	0.05	0.48	0.21	0.40	0.11	0.46	0.23	0.04	-0.09	0.53	0.12	-0.31	1.00	0.49	-0.10	0.21	-0.16	-0.31	-0.40	0.18	-0.52	-0.14	-0.54	-0.20	-0.20	0.08	-0.12
UAC	-0.23	0.31	0.35	0.12	0.09	0.14	0.11	0.29	-0.13	0.32	0.58	0.23	0.33	0.33	0.18	0.14	-0.61	0.49	1.00	-0.38	0.07	-0.13	-0.13	-0.32	-0.03	-0.09	-0.17	-0.41	-0.03	-0.11	0.04	0.07
CRE	0.28	-0.06	-0.31	0.09	-0.10	-0.15	-0.06	-0.52	0.36	-0.55	-0.24	-0.08	-0.15	-0.33	-0.12	-0.06	0.34	-0.10	-0.38	1.00	0.17	0.17	0.16	0.29	0.23	0.24	0.36	0.31	-0.09	0.46	-0.01	-0.20
SHP	0.06	0.06	0.20	0.10	0.11	-0.09	0.17	0.12	0.23	0.08	0.05	-0.25	0.13	-0.13	-0.15	-0.25	-0.17	0.21	0.07	0.17	1.00	-0.04	0.28	0.10	0.37	0.19	0.14	-0.01	-0.21	0.26	-0.17	-0.06
K	0.09	0.17	-0.03	0.23	0.23	0.22	0.21	-0.07	0.09	-0.09	0.03	0.06	-0.19	-0.03	0.16	0.11	0.10	-0.16	-0.13	0.17	-0.04	1.00	-0.01	0.45	0.00	0.19	0.20	0.24	0.09	0.13	0.25	-0.32
Na	0.23	-0.23	-0.34	0.22	-0.10	0.22	-0.19	-0.07	0.00	-0.07	-0.31	-0.15	-0.02	-0.01	-0.30	-0.28	0.13	-0.31	-0.13	0.16	0.28	-0.01	1.00	0.24	0.39	0.24	0.46	0.37	-0.03	0.27	0.00	0.03
CL	0.19	-0.04	-0.23	0.21	-0.10	0.08	-0.16	-0.08	-0.05	-0.03	-0.26	-0.12	-0.11	0.17	-0.13	-0.28	0.04	-0.40	-0.32	0.29	0.10	0.45	0.24	1.00	-0.06	0.42	0.48	0.52	-0.11	0.22	0.10	-0.02

Ca	0.14	0.26	0.13	-0.06	0.02	-0.12	0.12	-0.05	0.48	-0.17	-0.08	0.00	0.07	0.08	-0.11	0.11	0.13	0.18	-0.03	0.23	0.37	0.00	0.39	-0.06	1.00	-0.19	0.30	0.01	-0.01	0.31	-0.03	-0.06
P	0.06	-0.07	-0.39	0.47	-0.40	-0.12	-0.43	-0.32	-0.20	-0.25	-0.09	0.05	0.23	0.25	-0.56	-0.13	0.03	-0.52	-0.09	0.24	0.19	0.19	0.24	0.42	-0.19	1.00	0.43	0.33	0.15	0.38	0.22	-0.05
Mg	0.29	0.13	-0.10	0.33	-0.26	-0.12	-0.24	-0.42	0.23	-0.44	-0.26	0.12	0.22	0.38	-0.15	0.18	-0.02	-0.14	-0.17	0.36	0.14	0.20	0.46	0.48	0.30	0.43	1.00	0.44	0.06	0.25	0.22	-0.26
CYS	0.43	-0.44	-0.44	0.17	-0.36	-0.22	-0.34	-0.42	-0.04	-0.36	-0.45	-0.36	-0.20	0.11	-0.28	-0.04	0.14	-0.54	-0.41	0.31	-0.01	0.24	0.37	0.52	0.01	0.33	0.44	1.00	0.02	0.42	0.12	-0.02
CK	-0.13	-0.07	-0.03	0.11	0.05	-0.26	0.17	-0.23	0.06	-0.23	0.22	0.55	0.01	0.34	0.03	0.26	0.34	-0.20	-0.03	-0.09	-0.21	0.09	-0.03	-0.11	-0.01	0.15	0.06	0.02	1.00	0.09	0.19	-0.11
WBC	0.47	-0.23	-0.22	0.20	-0.45	-0.38	-0.35	-0.34	0.00	-0.30	-0.07	-0.01	0.13	-0.07	-0.40	0.07	0.25	-0.20	-0.11	0.46	0.26	0.13	0.27	0.22	0.31	0.38	0.25	0.42	0.09	1.00	0.11	-0.06
RBC	-0.01	0.04	-0.14	-0.08	0.22	0.01	0.26	-0.21	0.25	-0.29	0.12	0.45	-0.20	0.03	0.14	0.24	0.36	0.08	0.04	-0.01	-0.17	0.25	0.00	0.10	-0.03	0.22	0.22	0.12	0.19	0.11	1.00	0.01
PLT	-0.10	0.02	0.10	-0.13	-0.17	-0.06	-0.20	0.18	-0.02	0.18	-0.07	-0.12	-0.11	0.05	-0.11	-0.21	-0.01	-0.12	0.07	-0.20	-0.06	-0.32	0.03	-0.02	-0.06	-0.05	-0.26	-0.02	-0.11	-0.06	0.01	1.00

Note: Values represent Spearman's rank correlation coefficients. Bold values indicate |r| ≥ 0.6. Abbreviations: TG, triglycerides; CHO, total cholesterol; HD-CHO, high-density lipoprotein cholesterol; LD-CHO, low-density lipoprotein cholesterol; BIL, total bilirubin; DBIL, direct bilirubin; IBIL, indirect bilirubin; PRO, total proteins; ALB, albumin; GLO, globulin; AST, aspartate aminotransferase; ALT, alanine aminotransferase; GT, γ-glutamyl transpeptidase; ALP, alkaline phosphatase; CLT, cholinesterase; TBC, total bile acid; GLC, glucose; UAM, urea; UAC, uric acid; CRE, creatinine; SHP, serum hydrogen phosphate; K, potassium; Na, sodium; CL, chlorine; Ca, calcium; P, phosphorus; Mg, magnesium; CYS, homocysteine; CK, creatine kinase; WBC, white blood cell count; RBC, red blood cell count; PLT, platelet count.

Table S3 The description of the overlap genes from three selection methods.

Gene	Position	Tajiama'D	Pi	CLR
Tajima'D & θπ & CLR overlap gene				
CYM	3:32950001-33000000	-2.1377	3.16977	237.9477
PROK1	3:32950001-33000000	-2.1377	3.16977	237.9477
SPATA1	3:59500001-59550000	-2.27287	3.38926	190.1008
GNG5	3:59550001-59600000	-2.4336	3.35948	190.1008
CTBS	3:59500001-59550000	-2.27287	3.38926	85.63211
REV3L	9:39000001-39050000	-2.0664	3.45225	98.74975
SLC16A10	9:39200001-39250000	-1.88311	3.3561	40.69765
BOLA-DQB	23:25600001-25650000	1.70948	3.86333	398.5323
BOLA-DQA5	23:25600001-25650000	1.70948	3.86333	398.5323
Tajima'D & θπ overlap gene				
SUMO1	2:90750001-90800000	-1.88224	3.28897	*
DNM3	16:39850001-39900000	-0.74006	3.24427	*
ZNF783	4:112350001-112400000	-2.11654	3.25731	*
DCLK3	22:10750001-10800000	-2.28749	3.64626	*
SYN3	5:71600001-71650000	-0.61355	3.12426	*
BMPR2	2:90850001-90900000	-2.03568	3.46708	*
ZNF212	4:112300001-112350000	-2.4226	3.35584	*
OTUD4	17:13150001-13200000	-2.32598	3.49431	*
ADGRL3	6:77050001-77100000	-1.87499	3.1429	*
RAB11FIP2	26:38300001-38350000	-2.08566	3.47784	*
AKNA	8:103600001-103650000	-2.29773	3.15458	*
LARP1B	17:29600001-29650000	-1.79927	3.13958	*
NOP58	2:90800001-90850000	-1.76679	3.34051	*
ARHGAP44	19:31250001-31300000	1.52984	3.3675	*
MDN1	9:60400001-60450000	-2.04522	3.27615	*
CDK19	9:39500001-39550000	-2.50188	3.60645	*
ABCE1	17:13150001-13200000	-2.32598	3.49431	*
RALGAPA1	21:45800001-45850000	-1.79538	3.28293	*
GTF3C6	9:39350001-39400000	-2.30454	3.45289	*
ZNF282	4:112300001-112350000	-2.4226	3.35584	*
WWP2	18:36900001-36950000	-2.12966	3.28355	*
MFNG	5:75850001-75900000	-1.97476	3.34123	*
TRANK1	22:10650001-10700000	-2.3541	3.29071	*
DNAJC18	7:50650001-50700000	0.72081	3.1966	*
GOLGA4	22:10800001-10850000	-2.20184	3.77309	*
WHRN	8:103650001-103700000	-2.42375	3.2677	*
AMD1	9:39400001-39450000	-2.55133	3.70875	*
CASP8AP2	9:60400001-60450000	-2.04522	3.27615	*
ANAPC10	17:13250001-13300000	-2.57014	3.67257	*
NAA35	8:79200001-79250000	-1.87224	3.25641	*
RPF2	9:39350001-39400000	-2.30454	3.45289	*

θπ & CLR overlap gene				
GFOD2	18:35250001-35300000	*	3.22307	27.87009
ATP6V0D1	18:35050001-35100000	*	3.20729	29.78581
SLC4A8	5:28300001-28350000	*	3.25294	114.6444
RIPOR1	18:35100001-35150000	*	3.30645	29.78581
ABL2	16:60550001-60600000	*	3.13541	25.83455
SYT10	5:76350001-76400000	*	3.29915	69.7416
CCDC91	5:81300001-81350000	*	3.15418	31.18766
BTBD11	5:70600001-70650000	*	3.12401	36.88924
AGRP	18:35050001-35100000	*	3.20729	29.78581
ACAD11	1:136900001-136950000	*	3.33945	32.51168
PROX1	16:69550001-69600000	*	3.17349	201.5837
RANBP10	18:35300001-35350000	*	3.22308	27.87009
ACKR4	1:136950001-137000000	*	3.18388	32.51168
SDK2	19:57800001-57850000	*	3.35051	33.5934
TSNAXIP1	18:35300001-35350000	*	3.22308	27.87009
Tajima'D & CLR overlap gene				
SCN8A	5:28050001-28100000	2.75478	*	114.6444
FAM181A	21:58500001-58550000	-1.97934	*	43.75042
ASB2	21:58500001-58550000	-1.97934	*	43.75042

Table S4 KEGG pathway enrichment analysis of candidate genes under selection in Yunling cattle.

Pathway	Pvalue	Count
Allograft_rejection	1.76E-05	11
Asthma	1.76E-05	10
Graft-versus-host_disease	2.93E-05	10
Intestinal_immune_network_for_IgA_production	6.05E-05	11
Autoimmune_thyroid_disease	1.29E-04	11
Type_I_diabetes_mellitus	1.33E-04	10
Viral_myocarditis	2.21E-04	11
Inflammatory_bowel_disease_(IBD)	5.63E-04	11
Staphylococcus_aureus_infection	7.73E-04	10
HTLV-I_infection	0.001882624	21
Cell_adhesion_molecules_(CAMs)	0.002005963	15
Leishmaniasis	0.005190891	10
Rheumatoid_arthritis	0.005475017	11
Antigen_processing_and_presentation	0.007465143	10
Phagosome	0.030081557	13
Herpes_simplex_infection	0.053024681	14
Toxoplasmosis	0.082497097	10
Tuberculosis	0.239840916	12
Systemic_lupus_erythematosus	0.249242714	10
Fanconi_anemia_pathway	0.250885731	6
Influenza_A	0.427152068	11
TGF-beta_signaling_pathway	0.440164789	7
Cell_cycle	0.817302875	8
Bacterial_invasion_of_epithelial_cells	0.817302875	6
Hippo_signaling_pathway	0.817302875	9

Table S5 Genome-wide association analysis for Albumin traits

Chromosome	Position	P_{wald}	Interval	Gene
4	22612810	6.53E-07	intronic	*DGKB*
**6**	**86252896**	**4.55E-08**	**intronic**	***MOB1B***
**6**	**86252905**	**4.55E-08**	**intronic**	***MOB1B***
**6**	**86252932**	**3.15E-08**	**intronic**	***MOB1B***
6	86321027	2.52E-07	intronic	*DCK*
6	86324614	5.48E-07	intronic	*DCK*
6	86325846	1.64E-07	intronic	*DCK*
6	86333056	7.52E-07	intronic	*DCK*
6	86339450	1.33E-07	intronic	*DCK*
6	86339465	2.33E-07	intronic	*DCK*
6	86339472	3.03E-07	intronic	*DCK*
6	86339727	3.49E-07	intronic	*DCK*
9	9841168	4.68E-07	intronic	*SMAP1*
10	83198164	7.50E-07	intronic	*SIPA1L1*
11	32475703	7.30E-07	intronic	*NRXN1*
13	80637838	4.30E-07	intronic	*TSHZ2*
15	30765568	4.43E-07	intergenic	*TRIM29* (dist=53308), *OAF* (dist=18023)
15	58307261	9.24E-07	intergenic	*LIN7C* (dist=82708), *BDNF* (dist=78010)
19	52452378	4.65E-07	intronic	*CARD14*
19	52456263	2.23E-07	intronic	*CARD14*
20	61394161	1.53E-07	intronic	*CTNND2*
22	8038643	7.18E-07	intronic	*PDCD6IP*
28	28224853	9.25E-07	intronic	*ASCC1*

Note：The significant signals were pointed in bold in the table.

Table S6 Genome-wide association analysis for Cysteine traits

Chromosome	Position	P_{wald}	Interval	Gene
2	37571229	8.35E-07	intergenic	*PKP4*(dist=73136)
2	43711260	4.28E-07	intronic	*FMNL2*
2	127707139	8.13E-07	intergenic	*TMEM50A*(dist=3666), *RSRP1*(dist=27189)
3	13691810	6.35E-07	intergenic	*ETV3*(dist=38642)
7	24139933	7.21E-07	intronic	*CHSY3*
7	24140041	9.35E-08	intronic	*CHSY3*
11	31321669	8.30E-07	exonic	*FSHR*
13	7257445	6.72E-07	intergenic	*TASP1*(dist=6515), *ESF1*(dist=63645)
13	13140467	4.62E-07	intronic	*CELF2*
15	44262111	7.28E-07	intergenic	*STK33*(dist=109774), *LMO1*(dist=19171)
17	55113583	8.13E-07	intergenic	*PHETA1*(dist=2536), *SH2B3*(dist=27178)
18	11374707	5.92E-07	intronic	*GSE1*
24	45110686	7.19E-07	intronic	*SLC14A2*
**25**	**9597173**	**3.41E-08**	**intronic**	***CIITA***
25	15658565	6.75E-07	intronic	*XYLT1*
25	24689745	3.09E-07	intergenic	*KDM8*(dist=88782)

Note：The significant signals were pointed in bold in the table.

Table S7 Genome-wide association analysis for WBC traits

Chromosome	Position	P_{wald}	Interval	Gene
2	1.34E+08	1.40E-07	intergenic	*TAS1R2*(dist=52674)
3	10511565	7.17E-07	intergenic	*ACKR1*(dist=14616)
10	12056058	7.61E-07	exonic	*UBAP1L*
12	21025221	1.68E-07	intronic	*WDFY2*
12	21026519	9.43E-07	intronic	*WDFY2*
12	21220670	5.81E-07	intronic	*DHRS12*
12	21286581	1.85E-07	intergenic	*DHRS12*(dist=40097), *CCDC70*(dist=16879)
12	21300993	5.68E-07	intergenic	*DHRS12*(dist=54509), *CCDC70*(dist=2467)
12	21329369	3.84E-07	intergenic	*CCDC70*(dist=19726), *ATP7B*(dist=11646)
12	21649965	8.94E-07	intergenic	*VPS36*(dist=2983), *THSD1*(dist=1403)
16	2762564	6.14E-07	intronic	*NFASC*
16	22794096	8.62E-07	intergenic	*LYPLAL1*(dist=103416)
18	34039557	6.14E-07	intergenic	*CDH5*(dist=83164)
18	52728197	8.75E-07	intronic	*CLASRP*
18	52735141	8.79E-07	intronic	*CLASRP*
18	53590668	3.25E-07	downstream	*HIF3A*
20	67580476	4.86E-07	intergenic	*ICE1*(dist=71805)
**23**	**27871589**	**3.403E-08**	**intronic**	***BOLA***
**23**	**25617491**	**2.10E-08**	**intronic**	***BOLA-DQB***
**23**	**25637963**	**2.10E-08**	**intronic**	***BOLA-DQA5***
28	40454785	5.99E-07	intronic	*GRID1*

Note：The significant signals were pointed in bold in the table.

Table S8 Genome-wide association analysis for Bilirubin traits

Chromosome	Position	P_{wald}	Interval	Gene
3	81064713	5.24E-07	intergenic	*CACHD1*(dist=41760), *UBE2U*(dist=180218)
4	36449190	9.43E-07	intronic	*SEMA3A*
4	40484445	1.93E-07	intergenic	*CD36*(dist=10389), *GNAT3*(dist=89840)
**4**	**43058322**	**3.21E-08**	**intronic**	***MAGI2***
4	43856314	2.90E-07	intronic	*GSAP*
4	56453856	7.09E-07	intronic	*DOCK4*
4	63396712	5.90E-07	intronic	*BBS9*
4	73403427	5.45E-08	intronic	*ZNF804B*
5	35711614	3.33E-07	intronic	*NELL2*
5	47114895	6.37E-08	intronic	*GRIP1*
5	49242925	1.28E-07	intergenic	*RASSF3*(dist=52018), *TBK1*(dist=29035)
5	53062651	1.79E-07	intergenic	*SLC16A7*(dist=634478)
5	79674703	3.07E-08	intergenic	*IPO8*(dist=752343), *TMTC1*(dist=133167)
5	79744500	8.02E-07	intergenic	*IPO8*(dist=822140), *TMTC1*(dist=63370)
5	79794832	1.61E-07	intergenic	*IPO8*(dist=872472), *TMTC1*(dist=13038)
**5**	**79794843**	**7.07E-08**	**intergenic**	***IPO8*(dist=872483), *TMTC1*(dist=13027)**
5	79796092	1.22E-07	intergenic	*IPO8*(dist=873732), *TMTC1*(dist=11778)
5	79810228	8.53E-07	intronic	*TMTC1*
7	23283555	7.83E-07	intergenic	*HINT1*(dist=5007)
8	107456443	7.81E-07	intergenic	*TLR4*(dist=83646)
**9**	**76284878**	**5.19E-10**	**intronic**	***NHSL1***
12	76362799	2.67E-07	intronic	*TM9SF2*
12	76381434	3.34E-07	intronic	*TM9SF2*
13	6667773	2.25E-07	intronic	*SPTLC3*
13	16496054	7.83E-07	intronic	*SFMBT2*
**13**	**59072619**	**9.12E-08**	**intergenic**	***BMP7*(dist=97573)**
16	65016274	9.29E-07	intronic	*COLGALT2*
18	5411400	3.10E-07	intronic	*WWOX*
18	5412233	6.50E-07	intronic	*WWOX*
19	40777200	3.66E-07	exonic	*CCR7*
25	23930219	7.74E-07	intergenic	*HS3ST4*(dist=133472)
25	25504551	1.52E-07	intronic	*GSG1L*
26	4165165	6.90E-07	intergenic	*PCDH15*(dist=344105)

29	23636315	4.96E-07	intronic	*NELL1*

Note：The significant signals were pointed in bold in the table.

Table S9 Genome-wide association analysis for Indirect bilirubin traits

Chromosome	Position	P_{wald}	Interval	Gene
2	72284606	8.85E-07	intergenic	*GLI2*(dist=277073)
2	72288322	7.80E-07	intergenic	*GLI2*(dist=273357)
2	95145700	7.67E-07	intergenic	*FASTKD2*(dist=60688)
4	36449190	1.66E-07	intronic	*SEMA3A*
4	36449212	3.64E-07	intronic	*SEMA3A*
4	36450588	1.34E-07	intronic	*SEMA3A*
4	40484445	1.87E-07	intergenic	*CD36*(dist=10389), *GNAT3*(dist=89840)
4	43856314	5.53E-07	intronic	*GSAP*
**4**	**46076019**	**2.99E-08**	**intronic**	***LHFPL3***
5	47092130	5.25E-07	intronic	*GRIP1*
5	47097230	6.82E-07	intronic	*GRIP1*
5	47114895	1.75E-07	intronic	*GRIP1*
5	47319491	8.20E-07	intronic	*GRIP1*
5	47342888	4.39E-07	intronic	*GRIP1*
5	47481390	4.19E-07	UTR3	*HELB2*
5	49199880	5.24E-07	intergenic	*RASSF3*(dist=8973), *TBK1*(dist=72080)
5	49242925	6.18E-07	intergenic	*RASSF3*(dist=52018), *TBK1*(dist=29035)
5	49325784	2.01E-07	intronic	*XPOT*
**5**	**49325799**	**3.45E-08**	**intronic**	***XPOT***
5	51293612	9.52E-07	intergenic	*USP15*(dist=17822), *FAM19A2*(dist=67091)
**5**	**53062651**	**4.62E-08**	**intergenic**	***SLC16A7*(dist=634478)**
5	53086719	1.42E-07	intergenic	*SLC16A7*(dist=610410)
5	79674703	2.71E-07	intergenic	*IPO8*(dist=752343), *TMTC1*(dist=133167)
5	79794843	9.37E-07	intergenic	*IPO8*(dist=872483), *TMTC1*(dist=13027)
8	36531774	7.77E-07	intronic	*PTPRD*
8	107456443	8.60E-07	intergenic	*TLR4*(dist=83646),
**9**	**76284878**	**3.40E-09**	**intronic**	***NHSL1***
12	76362799	2.93E-07	intronic	*TM9SF2*
13	6667773	5.78E-07	intronic	*SPTLC3*
13	59072619	5.08E-07	intergenic	*BMP7*(dist=97573)
16	5326817	2.96E-07	intergenic	*CD55*(dist=47423), *CR2*(dist=70548)
16	66244669	6.94E-07	intronic	*SWT1*
18	22952926	4.58E-07	intergenic	*IRX3*(dist=334516)
29	23636315	9.63E-07	intronic	*NELL1*

Note：The significant signals were pointed in bold in the table.

Table S10 Genome-wide association analysis for Low-density lipoprotein cholesterol traits

Chromosome	Position	P_{wald}	Interval	Gene
1	63473628	7.27E-07	intergenic	*IGSF11*(dist=274521)
1	76667184	3.49E-07	intronic	*IL1RAP*
1	77440999	1.38E-07	intronic	*TP63*
1	77443822	4.94E-07	intronic	*TP63*
1	83384513	2.53E-07	intronic	*YEATS2*
1	93909508	7.23E-07	intronic	*NLGN1*
**1**	**96723402**	**1.63E-09**	**intergenic**	***RPL22L1*(dist=146581), *SLC7A14*(dist=141666)**
1	96723508	1.89E-07	intergenic	*RPL22L1*(dist=146687), *SLC7A14*(dist=141560)
**1**	**96723777**	**4.57E-09**	**intergenic**	***RPL22L1*(dist=146956), *SLC7A14*(dist=141291)**
2	5645582	4.28E-07	intronic	*NAB1*
2	25181578	8.39E-07	intergenic	*TLK1*(dist=38345)
2	32197911	2.80E-07	intergenic	*GRB14*(dist=387265), *FIGN*(dist=427706)
2	39102090	5.22E-07	intergenic	*GALNT5*(dist=32439), *TRNAC-GCA*(dist=197134)
2	40651541	6.47E-07	intergenic	*KCNJ3*(dist=529228)
2	47892592	1.13E-07	intergenic	*MBD5*(dist=39278), *PTMA*(dist=3461)
**2**	**59383227**	**4.08E-08**	**intergenic**	***THSD7B*(dist=127521)**
2	60481101	7.70E-07	intergenic	*THSD7B*(dist=59287), *TRNAC-GCA*(dist=233723)
2	96592681	2.94E-07	intronic	*PIKFYVE*
3	4740045	9.60E-07	intergenic	*PBX1*(dist=258934)
3	25183081	8.47E-07	intronic	*SPAG17*
3	42094667	2.37E-07	intergenic	*S1PR1*(dist=55343)
3	42096110	2.23E-07	intergenic	*S1PR1*(dist=56786)
3	91284499	8.52E-07	intronic	*USP24*
3	93126098	5.52E-07	intronic	*SLC1A7*
5	80011601	7.39E-07	intronic	*TMTC1*
5	80011918	5.36E-08	intronic	*TMTC1*
5	80011919	5.36E-08	intronic	*TMTC1*
5	80026144	6.81E-07	intronic	*TMTC1*
5	80037982	5.90E-07	intronic	*TMTC1*
6	91200255	5.33E-07	intronic	*FAM47E*
6	91206922	9.68E-07	ncRNA_exonic	*LOC107132577*
6	91991108	1.36E-07	intergenic	*CCNG2*(dist=2436)
7	2784550	4.69E-07	intergenic	*OBSCN*(dist=81008), *IBA57*(dist=171533)
7	2784571	3.76E-07	intergenic	*OBSCN*(dist=81029),

				IBA57(dist=171512)
7	2784574	3.20E-07	intergenic	*OBSCN*(dist=81032), *IBA57*(dist=171509)
7	2815175	4.76E-07	intergenic	*OBSCN*(dist=111633), *IBA57*(dist=140908)
7	2815196	4.76E-07	intergenic	*OBSCN*(dist=111654), *IBA57*(dist=140887)
7	3814575	7.17E-07	downstream	*GATAD2A*
7	3834526	1.18E-07	intronic	*GATAD2A*
10	55991919	1.27E-07	intronic	*UNC13C*
10	56078416	2.84E-07	intronic	*UNC13C*
10	63994223	1.77E-07	downstream	*LOC786643*
10	67842479	6.92E-07	UTR3	*ATG14*
11	75233067	1.29E-07	intergenic	*ATAD2B*(dist=19186)
**11**	**95947794**	**2.44E-08**	**intronic**	***SCAI***
11	96243983	6.05E-07	intergenic	*GAPVD1*(dist=29607)
12	22681784	9.26E-07	intergenic	*COG6*(dist=16591)
12	79529704	3.32E-08	intergenic	*TRNAF-GAA*(dist=318407)
13	23936184	1.52E-07	intergenic	*ARMC3*(dist=28533)
13	71021959	8.06E-07	intronic	*PTPRT*
14	56988222	2.88E-07	intronic	*ANGPT1*
14	60434895	5.93E-07	intronic	*RIMS2*
15	58292358	2.69E-07	intergenic	*LIN7C*(dist=67805), *BDNF*(dist=92913)
15	73301851	5.52E-07	intergenic	*API5*(dist=121953)
16	3707264	5.93E-07	intergenic	*RAB7B*(dist=21736)
**16**	**73176448**	**2.14E-08**	**intronic**	***SYT14***
21	16485787	8.94E-07	intronic	*AKAP13*
22	48916424	7.42E-07	intergenic	*DUSP7*(dist=84078), *RPL29*(dist=25611)
22	54590947	5.21E-08	intronic	*ATP2B2*
22	54605277	5.23E-08	intronic	*ATP2B2*
22	54605299	6.81E-08	intronic	*ATP2B2*
22	54605310	7.95E-08	intronic	*ATP2B2*
**22**	**54608791**	**4.48E-08**	**intronic**	***ATP2B2***
**22**	**54611735**	**3.00E-08**	**intronic**	***ATP2B2***
**22**	**54629224**	**4.08E-07**	**intronic**	***ATP2B2***
22	54647638	9.13E-07	intronic	*ATP2B2*
22	54647641	6.04E-07	intronic	*ATP2B2*
22	54649067	2.26E-07	intronic	*ATP2B2*
22	56794038	7.27E-07	ncRNA_intronic	*PPARG-TSEN2*
26	22410934	6.93E-07	intronic	*FBXW4*
26	23631998	6.10E-07	intronic	*CNNM2*
26	25504060	3.13E-07	intronic	*SORCS3*

26	42312248	2.60E-07	intronic	*HTRA1*
26	43668004	2.57E-07	intergenic	*CPXM2*(dist=35222)
27	1687937	4.44E-07	intergenic	*MYOM2*(dist=171354), *CSMD1*(dist=461197)
27	2528305	9.77E-07	intronic	*CSMD1*
27	2528766	4.66E-07	intronic	*CSMD1*
27	2530630	5.47E-08	intronic	*CSMD1*
27	2532012	5.80E-07	intronic	*CSMD1*
27	2535476	1.85E-07	intronic	*CSMD1*
27	2539466	2.44E-07	intronic	*CSMD1*
27	2545038	8.62E-07	intronic	*CSMD1*
27	2546299	3.20E-07	intronic	*CSMD1*
27	2546304	3.20E-07	intronic	*CSMD1*
27	2546323	3.20E-07	intronic	*CSMD1*
27	2549982	4.16E-07	intronic	*CSMD1*
27	2551199	7.15E-07	intronic	*CSMD1*
27	2560600	3.02E-07	intronic	*CSMD1*
27	2564384	6.78E-08	intronic	*CSMD1*
27	2573931	8.90E-07	intronic	*CSMD1*

Note：The significant signals were pointed in bold in the table.

Table S11 Genome-wide association analysis for red cell distribution width traits

Chromosome	Position	P_{wald}	Interval	Gene
**1**	**60786198**	**5.16E-09**	**intronic**	***LSAMP***
**1**	**60786218**	**5.16E-09**	**intronic**	***LSAMP***
2	11881658	8.99E-07	intronic	*ZNF804A*
2	121302170	1.84E-07	intergenic	*CLDN12*(dist=7540)
2	127469305	6.28E-07	intergenic	*STMN1*(dist=19881), *PAQR7*(dist=7118)
3	33769337	7.23E-08	intergenic	*GSTM1*(dist=3481), *AMPD2*(dist=48946)
**3**	**34607039**	**3.29E-09**	**intronic**	***STXBP3***
3	85834882	8.14E-07	intergenic	*C3H1orf87*(dist=48177)
4	117072899	8.23E-07	intronic	*DPP6*
**5**	**7813740**	**3.92E-10**	**intergenic**	***NAV3*(dist=130007)**
5	76095673	1.59E-07	intergenic	*USP18*(dist=77372), *ALG10*(dist=11081)
5	87012465	7.37E-08	intergenic	*SOX5*(dist=381253)
6	29019915	6.75E-07	intronic	UNC5C
6	69347389	1.75E-07	intergenic	*LNX1*(dist=153302)
6	114541521	2.89E-07	intronic	*SH3TC1*
6	114549479	2.89E-07	intronic	*SH3TC1*
6	114556975	1.16E-07	intronic	*SH3TC1*
7	53860887	8.59E-07	intronic	*ARHGAP26*
**8**	**19333571**	**3.24E-08**	**intergenic**	***TUSC1*(dist=755844), *IZUMO3*(dist=300641)**
8	26554343	7.96E-07	intergenic	*ADAMTSL1*(dist=93027), *SH3GL2*(dist=44160)
8	37663677	5.04E-07	intergenic	*DMAC1*(dist=566316)
8	81475525	3.39E-07	intronic	*C8H9orf3*
9	98242806	3.03E-07	intronic	*PACRG*
10	86743362	5.37E-07	intergenic	*BATF*(dist=7251), *FLVCR2*(dist=20074)
11	10294290	6.12E-07	intergenic	*DCTN1*(dist=25848), *BCL-XLP2*(dist=12604)
11	1.04E+08	2.55E-07	intronic	*NOTCH1*
12	16646721	9.24E-07	intronic	*LRCH1*
12	17533423	5.41E-08	intergenic	*SUCLA2*(dist=275904)
12	17533424	5.41E-08	intergenic	*SUCLA2*(dist=275903)
12	52913412	8.97E-07	intergenic	*MIR2284S*(dist=10282), *TRNAC-GCA*(dist=78560)
12	73835409	1.96E-07	intronic	*HS6ST3*
**13**	**3747195**	**2.02E-08**	**intronic**	***SLX4IP***
13	3747202	8.65E-08	intronic	*SLX4IP*
13	3777908	6.36E-08	intronic	*SLX4IP*

13	12000185	6.02E-08	intronic	*CAMK1D*
**13**	**12000200**	**4.99E-08**	**intronic**	***CAMK1D***
**13**	**82172037**	**5.36E-09**	**intergenic**	***DOK5*(dist=127509)**
**13**	**82198804**	**3.36E-09**	**intergenic**	***DOK5*(dist=154276)**
15	51547173	8.59E-07	intronic	*TRPC2*
15	51547175	8.59E-07	intronic	*TRPC2*
16	28832947	4.06E-07	upstream	*EPHX1*
16	56409240	1.39E-07	intronic	*KIAA0040*
16	80019798	2.12E-07	intronic	*KDM5B*
16	80019826	2.12E-07	intronic	*KDM5B*
17	4835060	6.24E-07	intronic	*ARFIP1*
17	10632363	6.03E-07	intronic	*EDNRA*
17	67498812	7.76E-07	intronic	*TTC28*
17	68735634	1.58E-07	intronic	*NIPSNAP1*
18	6404829	2.23E-07	intronic	*MAF*
18	6404874	2.03E-07	intronic	*MAF*
18	6462636	5.67E-08	intronic	*MAF*
**18**	**6923993**	**1.11E-08**	**intergenic**	***DYNLRB2*(dist=401781)**
**18**	**6923999**	**1.11E-08**	**intergenic**	***DYNLRB2*(dist=401775)**
18	7133525	7.98E-08	intergenic	*DYNLRB2*(dist=192249)
18	7133530	7.98E-08	intergenic	*DYNLRB2*(dist=192244)
18	7223223	9.07E-08	intergenic	*DYNLRB2*(dist=102551)
**18**	**7353328**	**2.05E-09**	**intergenic**	***DYNLRB2*(dist=17439), *CDYL2*(dist=35997)**
18	9844405	7.28E-07	intronic	*CDH13*
19	8856029	5.48E-08	intergenic	*SRSF1*(dist=46221)
19	9364121	9.03E-08	intronic	*RNF43*
19	10422271	2.26E-07	intergenic	*YPEL2*(dist=55644), *DHX40*(dist=108729)
**19**	**13532356**	**3.89E-09**	**intronic**	***ACACA***
20	15901047	5.77E-07	intergenic	*HTR1A*(dist=311049)
20	43732955	3.10E-07	upstream	*TRNAC-GCA*
21	57213218	3.49E-07	intergenic	*SLC24A4*(dist=8478), *RIN3*(dist=19076)
21	57217863	7.23E-07	intergenic	*SLC24A4*(dist=13123), *RIN3*(dist=14431)
23	6629589	3.81E-07	intronic	*LRRC1*
23	23684182	8.28E-07	intergenic	*PKHD1*(dist=390700)
23	33078321	1.05E-07	intronic	*ACOT13*
24	7737821	5.41E-07	intronic	*DOK6*
24	9708815	1.68E-07	intergenic	*DSEL*(dist=245112), *CDH19*(dist=427176)
24	12609141	8.40E-07	intergenic	*SYT4*(dist=41128)
24	19882260	4.78E-07	intronic	*KIAA1328*

24	21621718	2.33E-07	intergenic	*INO80C*(dist=13564)
24	25314962	1.57E-07	intronic	*TRAPPC8*
25	16963696	3.25E-07	intronic	*TMC7*
28	16322227	8.31E-07	intronic	*ANK3*
28	33449881	4.97E-07	intergenic	*KCNMA1*(dist=62330), *DLG5*(dist=68830)

Note：The significant signals were pointed in bold in the table.

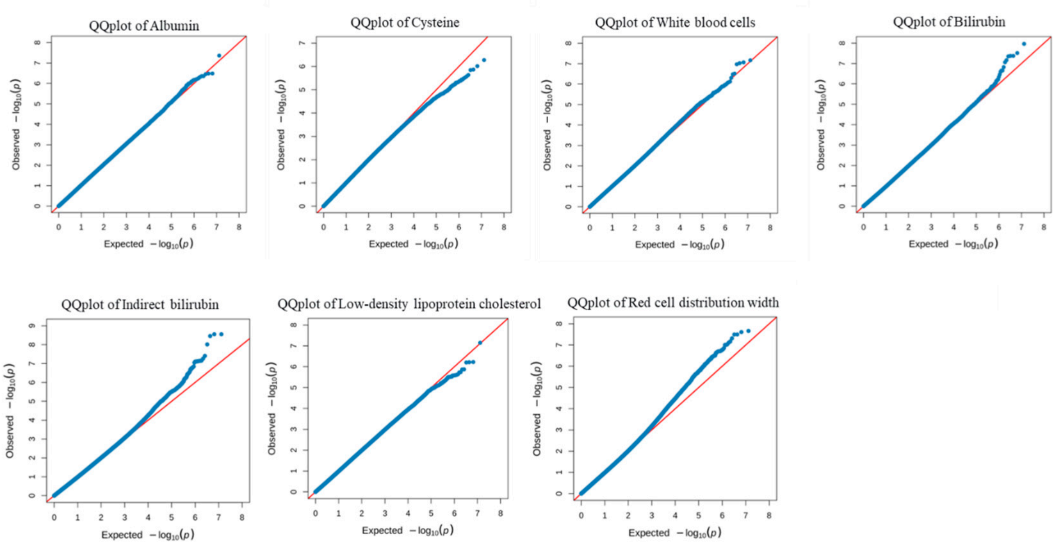

Figure S1. Quantile–quantile (QQ) plots for the seven traits with significant associations in the GWAS. (A) Albumin; (B) Cysteine; (C) White blood cell count; (D) Bilirubin; (E) Indirect bilirubin; (F) Low-density lipoprotein cholesterol; (G) Red cell distribution width. In each panel, the x-axis shows the expected $-\log_{10}(P)$ under the null hypothesis, and the y-axis shows the observed $-\log_{10}(P)$. The red line represents the expected distribution under the null hypothesis. The observed P values closely follow the expected null distribution across most of the range, with deviation only in the upper tail corresponding to the significant associations, indicating that the test statistics were adequately calibrated.
